# Proteomic changes of the bovine blood plasma in response to heat stress in a tropically adapted cattle breed

**DOI:** 10.3389/fgene.2024.1392670

**Published:** 2024-08-01

**Authors:** Henrique Goncalves Reolon, Natalya Gardezani Abduch, Ana Claudia de Freitas, Rafael Medeiros de Oliveira Silva, Breno de Oliveira Fragomeni, Daniela Lourenco, Fernando Baldi, Claudia Cristina Paro de Paz, Nedenia Bonvino Stafuzza

**Affiliations:** ^1^ Beef Cattle Research Center, Animal Science Institute, Sertãozinho, Brazil; ^2^ Department of Genetics, Ribeirao Preto Medical School (FMRP), University of Sao Paulo (USP), Ribeirão Preto, Brazil; ^3^ Agricultural Research Agency of the State of Minas Gerais (EPAMIG), Patos de Minas, Brazil; ^4^ Angus Genetics Inc., Saint Joseph, MO, United States; ^5^ Department of Animal Science, University of Connecticut, Storrs, CT, United States; ^6^ Department of Animal and Dairy Science, University of Georgia, Athens, GA, United States; ^7^ Department of Animal Science, School of Agricultural and Veterinary Sciences, Sao Paulo State University (UNESP), Jaboticabal, Brazil; ^8^ Sustainable Livestock Research Center, Animal Science Institute, São José do Rio Preto, Brazil

**Keywords:** beef cattle, *Bos taurus taurus*, Caracu, liquid chromatography-tandem mass spectrometry, pathways, potential biomarkers, thermotolerance

## Abstract

**Background:**

Identifying molecular mechanisms responsible for the response to heat stress is essential to increase production, reproduction, health, and welfare. This study aimed to identify early biological responses and potential biomarkers involved in the response to heat stress and animal’s recovery in tropically adapted beef cattle through proteomic analysis of blood plasma.

**Methods:**

Blood samples were collected from 14 Caracu males during the heat stress peak (HSP) and 16 h after it (heat stress recovery—HSR) assessed based on wet bulb globe temperature index and rectal temperature. Proteome was investigated by liquid chromatography-tandem mass spectrometry from plasma samples, and the differentially regulated proteins were evaluated by functional enrichment analysis using DAVID tool. The protein-protein interaction network was evaluated by STRING tool.

**Results:**

A total of 1,550 proteins were detected in both time points, of which 84 and 65 were downregulated and upregulated during HSR, respectively. Among the differentially regulated proteins with the highest absolute log-fold change values, those encoded by the *GABBR1, EPHA2, DUSP5, MUC2, DGCR8, MAP2K7, ADRA1A, CXADR, TOPBP1,* and *NEB* genes were highlighted as potential biomarkers because of their roles in response to heat stress. The functional enrichment analysis revealed that 65 Gene Ontology terms and 34 pathways were significant (*P* < 0.05). We highlighted those that could be associated with the response to heat stress, such as those related to the immune system, complement system, hemostasis, calcium, ECM-receptor interaction, and PI3K-Akt and MAPK signaling pathways. In addition, the protein–protein interaction network analysis revealed several complement and coagulation proteins and acute-phase proteins as important nodes based on their centrality and edges.

**Conclusion:**

Identifying differentially regulated proteins and their relationship, as well as their roles in key pathways contribute to improve the knowledge of the mechanisms behind the response to heat stress in naturally adapted cattle breeds. In addition, proteins highlighted herein are potential biomarkers involved in the early response and recovery from heat stress in tropically adapted beef cattle.

## 1 Introduction

Climate change directly affects beef cattle production, potentially leading to annual losses of up to 39 billion dollars by the end of the century, mainly in countries under tropical climates where temperatures are high and heat stress is more severe (Thornton et al., 2022).

Heat stress responses can promote several metabolic changes in an attempt to maintain body homeostasis, causing physiological, immunological, productive, and reproductive alterations, such as reduced activity of the reproductive and digestive systems as well as the immune system activation (Gonzalez-Rivas et al., 2020; McManus et al., 2022). Changes related to immune system functions may decrease food intake and increase disease susceptibility, affecting production and reproduction traits (McManus et al., 2020; McManus et al., 2022). Due to the widespread adoption of extensive production systems in most countries, beef cattle are one of the primary livestock species significantly affected by high environmental temperatures (Herbut et al., 2018).

The animal suffers heat stress when the amount of heat generated by its body exceeds the ability to dissipate heat into the environment (Abdelnour et al., 2019). Stress from an elevated body temperature can alter physiological, hematological, and hormonal functions (Abduch et al., 2022). Heat stress can lead to the intracellular synthesis of heat shock proteins in an attempt to protect the organism against stressful factors (Abdelnour et al., 2019; McManus et al., 2020), which can modify cellular responses, induce oxidative stress, cause metabolic and biochemical changes, and activate the necrosis and apoptosis pathways resulting in cell degradation and death (Kumar et al., 2018; Singh et al., 2018; Kim et al., 2022). Furthermore, several factors can affect the animal’s adaptability to heat stress, such as hair and coat characteristics, age, sex, and breed (Gaughan et al., 2019; Pires et al., 2019; Abduch et al., 2022).

Zebu cattle breeds (*Bos taurus indicus*) exhibit greater tolerance to tropical climatic conditions than taurine cattle breeds (*Bos taurus taurus*) (Sejian et al., 2018). Nevertheless, taurine breeds can also be selected to adapt to thermal stress, particularly Creole breeds that originate from regions with high environmental temperatures (Freitas et al., 2021; Saravanan et al., 2021). In this context, Caracu (*Bos taurus taurus*), a breed descendant from Iberian stock introduced by Portuguese settlers in the 16th century (Mercadante, 2005), is the largest Creole breed adapted to the Brazilian tropical climate used for beef production. Nowadays, Caracu is widely used in pure herds or crossing schemes since they can be more productive in their environments than exotic breeds, beyond its precocity, adaptability, rusticity, and good reproductive and productive performances (McManus et al., 2010; Lima et al., 2020; Pires et al., 2021; Pires et al., 2022).

Detection of thermotolerance biomarkers has become a hot topic in the last decade (Abdelnour et al., 2019; Kumar et al., 2023; Lemal et al., 2023). However, investigations are necessary to comprehend the fundamental mechanisms triggered by heat stress that contribute to its harmful impact on cattle, particularly at the proteomics levels, since post-transcriptional regulation of gene expression by miRNA exhibits an important role in cellular heat stress responses (Sengar et al., 2018). Proteomics involves the large-scale analysis of proteins, allowing for investigating quantities, varieties, roles, and interactions of all proteins in a given cell or tissue. The proteome of a given tissue is highly variable in response to environmental stimulation, resulting in a suitable tool for a comprehensive understanding of complex biological processes. The abundance of proteins circulating in the blood changes in response to environmental stressors providing valuable information about the nutritional, health, metabolic, and physiological conditions of animals and have been used to assess heat stress in cattle (Abdelnour et al., 2019; Gupta et al., 2022; Giannone et al., 2023; Kumar et al., 2023). In addition, changes in blood plasma proteins could be applied to discover potential biomarkers that can be useful indicators of animal’s physiological state since the blood plasma proteome interacts with all tissues through circulating factors (Min et al., 2016a; Cheng et al., 2018).

Increasing our knowledge of how naturally adapted breeds respond to heat stress and which proteins contribute to early response allows us to improve and develop strategies to reduce heat stress in animals under high environment temperatures. Thus, this study aimed to explore the early biological responses and pathways involved in the animal’s recovery from heat stress and to detect key proteins responsible for homeostasis in beef cattle through proteomic analysis of blood plasma.

## 2 Material and methods

### 2.1 Animals and data collection

All the experimental procedures were approved by the Institutional Animal Care and Use Committee at the Animal Science Institute (protocol code CEUA N°. 292–19, 7 October 2019), following guidelines for animal welfare (São Paulo State Law N°. 11.977). The experiment was carried out at the Beef Cattle Research Center of the Animal Science Institute (IZ), Sertãozinho, São Paulo, Brazil (21° 17′S and 48° 12′W), a subtropical region with warm and rainy summers and dry winters. The experiment was conducted in February (average temperature of 28.9°C and average humidity of 63.6%), one of the warmest months in this region.

A meteorological measurement base of a digital black globe thermometer ITEG-500 (Incon Eletronica Ltda, BR) was installed in the paddock where the experiment was performed to collect the environmental conditions. The wet bulb globe temperature (WBGT) index was used as an indicator of thermal comfort, which was calculated using the following equation (Parsons, 2006):

$$WBTG=0.7t_{nwb}+0.2t_{bg}+0.1t_{db}$$

where t_nwb_ is the natural wet bulb temperature obtained under sun and wind, t_bg_ is the black globe temperature, and t_db_ is the dry bulb temperature. Rectal temperature was also measured.

A total of 14 Caracu bulls were evaluated in this study. They weighed 423 ± 42.68 kg, were 16 months old, on average, belonged to the same contemporary group (farm and season or birth), and were reared on *Brachiaria sp*. forage pasture supplemented with mineral salt. All animals were kept in an unshaded paddock with food (60% sorghum silage, 13% soybean meal, 25% ground corn, 1.75% mineral salt, and 0.2% urea) and water *ad libitum* for 21 days before the blood collection.

The blood samples and rectal temperatures were collected from all animals in two time points to detect proteins involved in the early response to heat stress and animal’s recovery: 1) during heat stress peak (HSP): at 2:00 p.m., with the highest WBGT index (41.0) and the highest rectal temperatures recorded (mean of 40.5°C ± 0.32°C), indicating heat stress; 2) 16 h after heat stress peak (heat stress recovery—HSR): at 6:00 a.m., with the lowest WBGT index (19.3) and the lowest rectal temperatures recorded (mean of 37.3°C ± 0.21°C).

### 2.2 Proteomic analysis

A total of twenty-eight blood samples (14 during HSP and 14 during HSR) were centrifuged at 3,000 × g for 15 min at 15°C and 500 µL of plasma were immediately stored at −80°C prior to the liquid chromatography–tandem mass spectrometry (LC-MS/MS) analysis.

All plasma samples were submitted to albumin depletion with Cibacron Blue 3 GA Agarose (Sigma Aldrich, United States) packaged in Pierce™ screw cap spin columns (ThermoFisher Scientific, United States), according to the manufacturer’s instructions. Briefly, 10 µL of each plasma sample was diluted in 590 µL of Tris buffer (50 mM, pH = 8.0) and applied to the column. The eluent was collected and reapplied to the column for greater albumin retention. The collected samples represent plasma without the predominant presence of albumin (protein recovery of −32%). The Bradford method (Bradford, 1976) was used for protein quantification using the Protein Assay Dye Reagent Concentrate (Bio-Rad, United States), following the manufacturer’s instructions. The total protein mass of individual samples was estimated using a SpectraMax Plus 384 spectrophotometer (Molecular Devices, United States).

The electrophoretic profile of each sample was evaluated by SDS–PAGE. Briefly, each sample (−20 µg of proteins) was resuspended in XT Sample Buffer 1X (Bio-Rad, United States) with 20 µg of dithiothreitol (DTT) followed by incubation at 100°C for 5 min to break disulfide bonds and protein stabilization. Then, the samples were incubated in 100 µg iodoacetamide for 20 min for protein alkylation. The SDS-PAGE was performed with 12% Precise™ Protein Gels (Thermo Scientific, United States) at 40 V for 30 min, followed by 70 V for 2 h, using a Mini-PROTEAN II Electrophoresis System (Bio-Rad, United States). The gels were stained with GelCode™ Blue Stain Reagent (ThermoFisher Scientific, United States), according to the manufacturer’s recommendations.

The samples (50 µg of each) were prepared for MS/MS, as described in Tanamati et al. (2020), with some changes. The samples were incubated in DTT (1 mg/mg of protein) for 2 h at room temperature and then in iodoacetamide (3 mg/mg of protein) for 1 h at room temperature for disulfide bonds reduction and cysteine alkylation, respectively. The samples were diluted 5 X in ammonium bicarbonate solution (0.1 M, pH ≥ 8.0) to obtain a final volume of 500 µL. Then, the samples were incubated at 37°C overnight with 1 µg of trypsin and desalted using an Oasis HLB 1 cc vac cartridge (Waters, United States) following the manufacturer’s recommendations. The column was equilibrated with 5% acetonitrile solution with 0.1% formic acid, and the peptide fraction was eluted with 80% acetonitrile. Samples were dried in a SpeedVac (ThermoFisher Scientific, United States) concentrator, and 2 µg of each sample were evaluated, in duplicate, using an LTQ-Orbitrap ELITE mass spectrometer (Thermo-Finnigan, DE) coupled to a nanoflow LC–MS/MS system (Dionex Ultimate 3000 RLSCnano System, ThermoFisher Scientific, DE).

Peptides were fragmented using an analytical column (nanoEase MZ Peptide BEH C18, 130 Å, 1.7 µm × 75 μm x 250 mm, Waters) with a gradient from 4% to 50% of acetonitrile at a flow rate of 300 nL/min. The spectra data were obtained in an MS1 scan (m/z 375–1500, AGC target 1E6 ions, maximum ion injection time of 100 ms) at a resolution of 120,000. The most abundant ions were subjected to MS/MS (30% of collision energy, AGC target 1E5 ions, 1.2 m/z of isolation width, AGC target 1E5 ions, and resolution of 15,000).

Raw LC-MS/MS data were converted to mzXML, and the assignment of MS/MS spectra was performed using the open-source Comet algorithm v.2019 (Eng et al., 2013) and the bovine UniProt database (UniProt Consortium, 2023). The high-confidence peptide identification (FDR ≤0.05) and quantitation were obtained through the Peptide Prophet (Keller et al., 2002) and the XPRESS algorithm (Han et al., 2001), respectively. Peptides and their intensities were grouped to obtain protein intensity using a script in R software (R Core Team, 2021). Proteins with a fold-change ratio greater than 2 and less than 0.5 were considered upregulated and downregulated (*P* < 0.05), respectively.

The differentially regulated proteins were submitted to functional enrichment analysis using the DAVID v.2023q4 tool (Sherman et al., 2022), where Gene Ontology terms (biological processes and molecular functions) and pathways with *P* < 0.05 were considered significant. STRING v.12 (https://string-db.org/) was used for the protein–protein interaction (PPI) network analysis of proteins differentially regulated, with a high confidence score (0.7).

## 3 Results

### 3.1 Differentially regulated proteins

**FIGURE 1:**
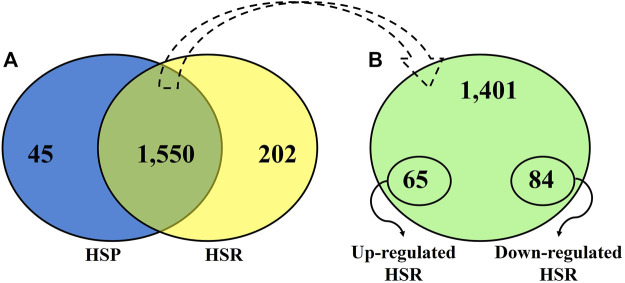
**(A)** Venn diagram showing the distribution of all proteins identified during heat stress peak (HSP) and heat stress recovery (HSR). **(B)**. Distribution of proteins identified in both time points (HSP and HSR).

After quality control, 45 proteins were identified only during HSP (Figure 1; Supplementary Table S1), and 202 proteins were detected only during HSR (Figure 1; Supplementary Table S2). A total of 1,550 proteins were detected in both periods, of which 84 and 65 proteins were significantly downregulated and upregulated during HSR, respectively (Figure 1; Supplementary Table S3). The top 20 differentially regulated proteins (with the highest absolute log-fold change values) between HSP and HSR are described in Table 1.

**TABLE 1:** Top 20 differentially regulated proteins detected in blood plasma samples during heat stress peak (HSP) and heat stress recovery (HSR).

UniProt ID	Protein	Gene symbol/Ensembl ID	Protein length	LogFC
A0A3Q1MBN0^a^	Gamma-aminobutyric acid type B receptor subunit 1	*GABBR1*	955	−9.073
E1BLN1^a^	Synaptonemal complex protein 1	*SYCP1*	915	−8.035
E1BMN7^a^	Ankyrin repeat and BTB/POZ domain-containing protein 2	*ABTB2*	1,191	−7.821
F1MQM7^a^	Dual specificity protein phosphatase 5	*DUSP5*	457	−6.798
A0A3Q1M2T0^a^	Mucin-2	*MUC2*	3,203	−6.175
Q28021^a^	Rho-associated protein kinase 2	*ROCK2*	1,388	−5.911
A0A3Q1N1L9^a^	F-box/LRR-repeat protein 19	*FBXL19*	1,020	−5.701
Q8WMV3^a^	Coxsackievirus and adenovirus receptor homolog	*CXADR*	365	−5.272
A0A3Q1LS24^a^	ATP-binding cassette sub-family B member 5	*ABCB5*	1,270	−4.741
A0A3Q1LQW4^a^	DNA topoisomerase 2-binding protein 1	*TOPBP1*	1,434	−4.739
F1MT60^a^	Nebulin	*NEB*	6,888	−4.342
Q28046^a^	Scinderin	*SCIN*	715	−4.328
F1N6W9^b^	Collagen type XVIII alpha 1 chain	*COL18A1*	1,514	4.176
E1B968^b^	Solute carrier family 7 member 2	*SLC7A2*	669	4.853
Q28146^b^	Neurexin-1	*NRXN1*	1,530	5.428
P18130^b^	Alpha-1A adrenergic receptor	*ADRA1A*	466	5.556
E1BJB4^b^	RPGRIP1 like	*RPGRIP1L*	1,270	5.622
A0A3Q1LMF7^b^	Mitogen-activated protein kinase kinase 7	*MAP2K7*	670	5.804
A6QR44^b^	Microprocessor complex subunit DGCR8	*DGCR8*	760	5.873
E1BJ31^b^	Ephrin type-A receptor 2	*EPHA2*	975	7.638

^a^ upregulated during HSP/downregulated during HSR.

^b^ downregulated during HSP/upregulated during HSR.

### 3.2 Functional enrichment analysis

**FIGURE 2:**
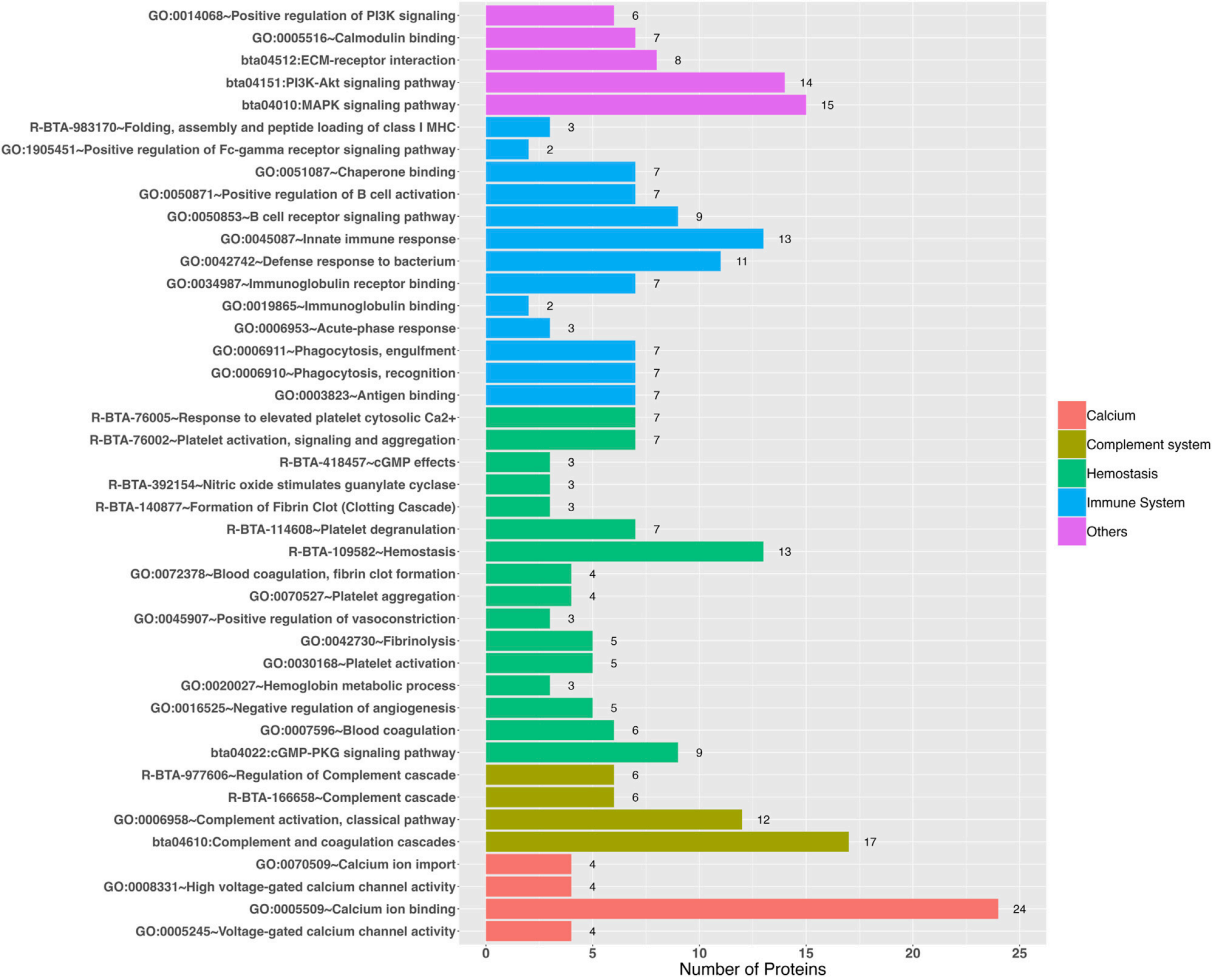
Gene Ontology terms and pathways revealed by functional enrichment analyses (*P* < 0.05) that could be involved in the response to heat stress.

The functional enrichment analysis revealed 38 Gene Ontology biological processes and 27 Gene Ontology Molecular Functions, as well as 19 REACTOME and 15 KEGG pathways as significant (Supplementary Table S4), in which those that could be related to heat stress were highlighted (Figure 2; Supplementary Figure S1), such as: 1) Immune system: immunoglobulin binding (GO:0019865), immunoglobulin receptor binding (GO:0034987), chaperone binding (GO:0051087), antigen binding (GO:0003823), antigen presentation: folding, assembly and peptide loading of class I MHC (R-BTA-983170), positive regulation of B cell activation (GO:0050871), B cell receptor signaling pathway (GO:0050853), innate immune response (GO:0045087), acute-phase response (GO:0006953), phagocytosis recognition (GO:0006910), phagocytosis engulfment (GO:0006911), and positive regulation of Fc-gamma receptor signaling pathway involved in phagocytosis (GO:1905451); 2) Complement system: complement activation classical pathway (GO:0006958), regulation of complement cascade (R-BTA-977606), terminal pathway of complement (R-BTA-166665), complement cascade (R-BTA-166658), and complement and coagulation cascades (bta04610); 3) Hemostasis: hemostasis (R-BTA-109582), blood coagulation (GO:0007596), blood coagulation, fibrin clot formation (GO:0072378), fibrinolysis (GO:0042730), formation of fibrin clot—clotting cascade (R-BTA-140877), hemoglobin metabolic process (GO:0020027), activation platelet activation (GO:0030168), platelet aggregation (GO:0070527), platelet activation, signaling and aggregation (R-BTA-76002), platelet degranulation (R-BTA-114608), response to elevated platelet cytosolic Ca2+ (R-BTA-76005), nitric oxide stimulates guanylate cyclase (R-BTA-392154), cGMP-PKG signaling pathway (bta04022), cGMP effects (R-BTA-418457), negative regulation of angiogenesis (GO:0016525), and positive regulation of vasoconstriction (GO:0045907); 4) Calcium: calcium ion import (GO:0070509), calcium ion binding (GO:0005509), high voltage-gated calcium channel activity (GO:0008331), and voltage-gated calcium channel activity (GO:0005245); 5) Calmodulin binding (GO:0005516); 6) ECM-receptor interaction (bta04512); 7) Phosphatidylinositol-3-kinase (PI3k): positive regulation of PI3K signaling (GO:0014068) and PI3K-Akt signaling pathway (bta04151); and 8) MAPK signaling pathway (bta04010).

### 3.3 Protein-protein interaction

**FIGURE 3:**
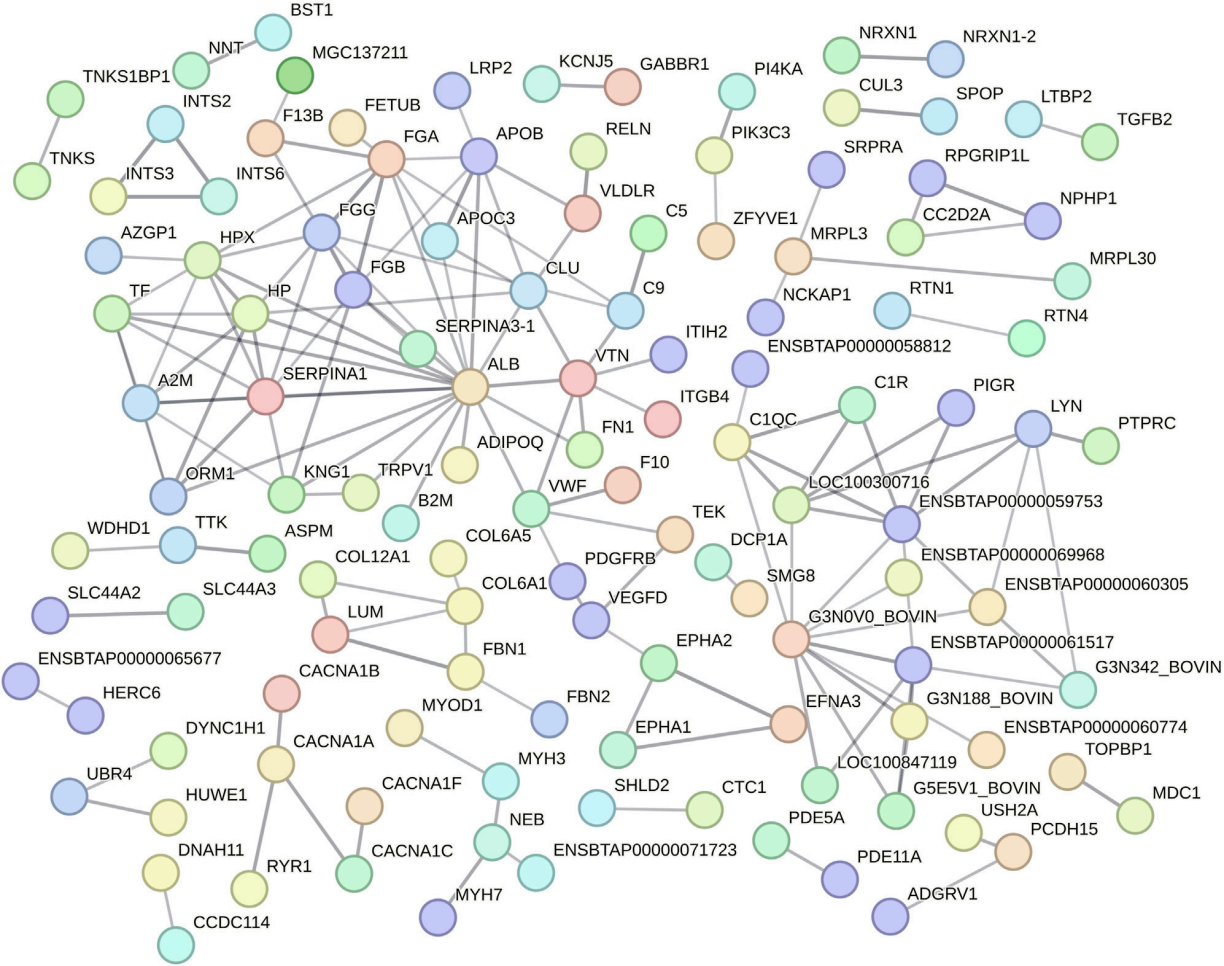
Protein-protein interaction analysis of all differentially regulated proteins between heat stress peak (HSP) and heat stress recovery (HSR). Nodes represent differentially expressed proteins (gene symbol) and lines between nodes refer to edges of confident interactions. Standalone nodes lacking edges were removed.

Function protein association network analysis of differentially regulated proteins encompassed 389 nodes and 158 interactions (edges), with an average local clustering coefficient of 0.239 and PPI enrichment *P* < 1.0e-16 (Figure 3; Supplementary Table S5). The differentially regulated proteins encoded by *ALB* (19 edges), *ENSBTAG00000048135* (10 edges), *FGA* (9 edges), *SERPINA1* (9 edges), *ENSBTAG00000047700* (8 edges), *FGG* (8 edges), *HP* (8 edges), *HPX* (8 edges), *A2M* (7 edges), *APOB* (7 edges), *FGB* (7 edges), *VTN* (7 edges), *CLU* (6 edges), *ENSBTAG00000051010* (6 edges), *LOC100300716* (6 edges), *TF* (6 edges), *C1QC* (5 edges), *KNG1* (5 edges), *LYN* (5 edges), *ORM1* (5 edges), *VWF* (5 edges), *APOC3* (4 edges), *C9* (4 edges), *COL6A1* (4 edges), and *ENSBTAG00000052621* (4 edges) genes were highlighted as important nodes based on their centrality and edges (Figure 3). Regarding its abundancy, 11 proteins highlighted by PPI analysis (encoded by *APOC3, C9, FGA, FGB, FGG, HP, KNG1, ORM1, VTN, VWF* and *TF* genes) had their abundancies increased during HSP. In contrast, six proteins had their abundancies increased during HSR (encoded by *ALB, APOB, C1QC, CLU, HPX,* and *SERPINA1* genes). In addition, alpha-2-macroglobulin (*A2M*) and tyrosine-protein kinase Lyn (*LYN*) were detected only during HSP, while collagen alpha-1(VI) chain (*COL6A1*) was detected only during HSR.

## 4 Discussion

### 4.1 Differentially regulated proteins

Among the top 20 differentially regulated proteins detected in this study, we highlighted those that could be related to response to heat stress (Table 1), as discussed below. *GABBR1* (Gamma-aminobutyric acid type B receptor subunit 1) encodes a receptor for a substantial inhibitory neurotransmitter in the central nervous system, the gamma-aminobutyric acid (GABA), which plays a crucial role in thermic stress reduction (Lee et al., 2023). Studies with *Gabbr1(−/−)* knockout mice have shown its role in body temperature regulation, locomotor activity, and behavior (Quéva et al., 2003; Haller et al., 2004; Jacobson et al., 2006). In the present study, we identified this receptor upregulated in the Caracu blood plasma during HSP, corroborating with Ramírez-Ayala et al. (2021) on its potential role in thermogenesis and its contribution to the adaptation of cattle to tropical conditions. The dual specificity protein phosphatase 5 (encoded by *DUSP5*) is a member of the dual specificity protein phosphatase subfamily, which negatively regulates members of the MAP kinase superfamily that acts in cellular proliferation and differentiation (Sonna et al., 2002). The dual specificity phosphatase 5 protein responds to hypo-osmotic stress in keratinocytes (Liovic et al., 2008). Studies in humans have detected that *DUSP5* expression increased by heat shock in human skin fibroblast (Ishibashi et al., 1994).

Mucin-2 (*MUC2*) is a glycoprotein produced by several epithelial tissues, which is mainly secreted from intestinal goblet cells and acts as an insoluble mucous protective barrier for the intestine, playing an important role in defense against inflammation and intestinal homeostasis (Ma et al., 2018). Studies have reported that heat stress increases mucin-2 secretion in the intestine (Pearce et al., 2014; Pearce et al., 2015), probably as a compensative mechanism to help maintain the protective barrier and structural integrity of this organ. The mucin-2 was also upregulated in the Caracu blood plasma during HSP.

Coxsackievirus and adenovirus receptor, encoded by *CXADR* gene, is predominantly produced on the surface of epithelial and endothelial cells, providing a barrier function and regulating the migration of immune cells. Tang et al. (2022) identified the *CXADR* gene downregulated in the ileum of pigs under heat stress. The DNA topoisomerase 2-binding protein 1 (*TOPBP1*) has been described as involved in DNA replication initiation and response to ionizing radiation. Tuul et al. (2013) reported that *TOPBP1* gene plays an essential role in heat tolerance since its downregulation drastically reduced cell viability upon hyperthermia. Nebulin (*NEB*) is a cytoskeletal matrix component that is essential to the structural and functional properties of skeletal muscle (Ottenheijm et al., 2012). Nebulin has been identified as significantly upregulated under moderate hyperthermia (39°C), promoting myofibrillogenesis (Guo et al., 2016). However, upon constant heat stress, nebulin was found downregulated (Hao et al., 2016).

The alpha-1A adrenergic receptor (*ADRA1A*) is a member of the G protein-coupled receptor superfamily, which regulates the growth and proliferation of several cells and has been associated with response to stress hormones (Chang et al., 1998). Dual specificity mitogen-activated protein kinase kinase 7 (*MAP2K7*) is involved in the signal transduction mediating the cell responses to proinflammatory cytokines and environmental stresses such as ultraviolet radiation and heat (Foltz et al., 1998; Chen et al., 2023). Microprocessor complex subunit DGCR8 (*DGCR8*), together with nuclear RNase III enzyme Drosha, mediates the biogenesis and processing of microRNAs (Faller et al., 2010). Knuckles et al. (2017) showed that acute heat stress causes radical relocation of microprocessor complex subunit DGCR8 to stress-induced genes, such as *HSP70*, which acts to co-transcriptionally mark mRNAs for subsequent degradation to control the response to heat stress. *DGCR8* gene has been associated with heat stress response in cattle (Otto et al., 2019; Luo et al., 2021). The *EPHA2* encodes the ephrin type-A receptor 2, which acts in many processes, including regulating blood vessel endothelial cell migration. Heat stress induces phosphorylation of the EPH receptor A2, which is known to signal via PI3K-AKT (Thompson et al., 2018).

### 4.2 Functional enrichment analysis

Several studies have reported the impact of the immune system and its inflammatory responses in cattle under heat stress (Min et al., 2016a; Min et al., 2016b; Sengar et al., 2018; Koch et al., 2019; Kumar et al., 2021). As reviewed by Cantet et al. (2021), individuals exposed to high environmental temperature redistribute their blood toward the periphery to dissipate heat, which causes a reduction in blood flow and oxygen, as well as nutrient supply to tissues. Metabolic adaptations lead to oxidative stress and the release of proinflammatory mediators that activate a systemic inflammatory response. Both innate and adaptive immunity can be affected by heat stress, where innate immunity is the first line of defense and interfaces with adaptive immunity, which is mediated by T and B lymphocytes providing immunological specificity and memory. Indeed, heat stress has been associated with reduced production of cytokines and immunoglobulins (Dahl et al., 2020), as well as lymphocyte proliferation and neutrophil phagocytosis (do Amaral et al., 2010; do Amaral et al., 2011), which contributes to the increased susceptibility to pathogens in cattle under heat stress (Cartwright et al., 2022).

Evolved as part of the innate immune system, the complement system is a proteolytic cascade in blood plasma that is fundamental to enhancing adaptive immune responses (Janeway et al., 2001). Proteins of the complement system react with one another and induce several inflammatory responses by recruiting inflammatory and immunocompetent cells that help fight infection through a nonspecific defense mechanism against pathogens (Janeway et al., 2001). Studies have described that heat stress alters the abundance of several proteins of the complement system in cattle blood plasma (Min et al., 2016a; 2017), reinforcing its important functions in the immune response and adaptation of animals under hot environments (Min et al., 2016a; Morenikeji et al., 2020; Yang et al., 2021; Skibiel et al., 2022).

Blood content may be directly or indirectly influenced by heat stress to contribute to reducing the deleterious effect of high temperature by activating body hemostasis mechanisms (Jo et al., 2021). Hemostasis involves a combination of blood clotting through converting fibrinogen (soluble) to the fibrin clot (insoluble) in sites of vascular injury to stop blood loss and subsequent dissolution of blood clots through fibrinolysis. Several interconnections have been described between the components of the complement, coagulation, and fibrinolysis systems, which share several serine proteinases with trypsin-like activity together with their regulators (Oikonomopoulou et al., 2012). Min et al. (2016a) described that heat stress increases many proteins of the coagulation system in cows’ blood plasma; this result was supported by several studies that highlight the coagulation system roles in the adaptation of animals under high temperatures (Min et al., 2016a; Morenikeji et al., 2020; Yang et al., 2021; Skibiel et al., 2022).

Hemostasis depends on the interaction between several proteins and cellular receptors, which are highly sensitive to changes in environmental temperature. High temperatures can disrupt molecular bonds that hold coagulation proteins in their secondary and tertiary structures (Levi, 2018). Platelets play an important role in hemostasis. Hyperthermia modulates platelet function, which could be affected via coagulation, inflammation, cytokines, and heat shock proteins. Platelet levels can also be regulated by interactions between leukocytes and endothelial cells induced by hyperthermia (Iba et al., 2023). Cyclic guanosine monophosphate (cGMP) is an important intracellular secondary messenger produced by guanylate cyclases that mediates the action of natriuretic peptides and nitric oxide, regulating many physiologic processes such as neurotransmission, platelet aggregation, vasodilation (Li et al., 2003), and ROS generation (Ferreira et al., 2015). Increasing intracellular levels of cGMP activates PKG, inhibiting platelet activation (Haslam et al., 1999). In addition, platelet responses to cGMP appear to have an early stimulatory response that promotes platelet activation, followed by a delayed inhibitory response that limits platelet aggregation (Li et al., 2003). Del Corvo et al. (2021) found the cGMP binding hypo-methylated in Nellore cattle compared to Angus cattle during heat stress, highlighting its essential role in the adaptation to heat stress. Studies with circRNAs (Zhang et al., 2023), mRNA (Kim et al., 2020; Liu et al., 2020), and miRNA (Liu et al., 2020) in blood samples of cows also have suggested an important role of cGMP-PKG signaling pathway in heat stress response.

Calmodulins are calcium binding proteins that plays important roles in several cellular processes, including regulation of the cell cycle, cell proliferation, apoptosis, signaling pathways, inflammation and the immune response. Evans and Tomasovic (1989) described the importance of calmodulin in hyperthermic cell killing and on the development of thermotolerance.

The extracellular matrix (ECM) comprises several structural and functional macromolecules that have important roles in maintaining the structure and function of both cell and tissue. Interactions between cells and the ECM, which are mediated by transmembrane molecules, control several cellular activities direct or indirect, such as proliferation, differentiation, migration, adhesion, and apoptosis. The ECM-receptor interaction (bta04512) pathway, which has a pivot role in hemostasis (Bergmeier and Hynes, 2012), been described as impacted by heat stress in goat (Mehaba et al., 2019), pig (Sarais et al., 2023) and fish (Zhou et al., 2023), being considered an essential adaptive pathway in cattle under hot environments (Morenikeji et al., 2020).

MAPK and PI3K-Akt signaling pathways have been described as highly influenced miRNA-mediated post-transcriptional regulation in response to heat stress in cattle (Sengar et al., 2018; Kumar et al., 2021). The mitogen-activated protein kinase (MAPK) signaling pathway (bta04010) plays an important role in the regulation of several cellular functions and physiological processes, such as cell proliferation, differentiation and migration, apoptosis, inflammation, innate immune defense, as well as response to many stressors such as ultraviolet irradiation, heat, ischemia, reactive oxygen species, cytokines, and osmotic shock (Muthusamy and Piva, 2010; Arthur and Ley, 2013; Yu et al., 2013). The inflammation triggered by heat stress may be carried out mainly through the MAPK signaling pathway (Fan et al., 2021), which promotes a protective effect against heat stress-induced cell apoptosis (Hao et al., 2018). MAPK signaling pathway has been identified as enriched in response to heat stress in many studies with several cattle breeds (Srikanth et al., 2017; Liu et al., 2020; Fang et al., 2021; Kumar et al., 2021).

Several types of cellular stimuli activate the PI3K-Akt signaling pathway (bta04151) and mediate essential cellular functions, such as cell proliferation, differentiation, growth and migration, as well as autophagy and apoptosis (Levine and Kroemer, 2008). Studies have reported that the PI3k/AKT pathway is activated by temperature increase (Yoshihara et al., 2013) and plays a critical role in the cellular metabolism and prevention of apoptosis induced by heat stress (Gao et al., 2013; Barrera et al., 2023). Several studies have reported the participation of the PI3K-Akt signaling pathway in response to heat stress in cattle (Srikanth et al., 2017; Del Corvo et al., 2021; Fang et al., 2021; Zhang et al., 2023).

### 4.3 Protein-protein interaction

Several proteins of complement and coagulation systems were highlighted as important nodes based on their centrality and edges, such as complement C1q subcomponent subunit C (*C1QC*) and alpha-1-antiproteinase (*SERPINA1*) that had their abundancies increased during HSR, in addition to complement component C9 (*C9*), fibrinogen alpha chain (*FGA*), fibrinogen beta chain (*FGB*), and fibrinogen gamma-B chain (*FGG*), which had their abundancies reduced during HSR. Min et al. (2016a) reported that heat stress alters plasma levels of complement system proteins, such as those reported herein, impairing immune function in lactating dairy cows under heat stress.

Several acute-phase proteins were also highlighted as essential nodes, including negative acute-phase proteins (albumin and transferrin), whose levels decrease during inflammation, as well as positive acute-phase proteins (clusterin, haptoglobin, hemopexin, and alpha-1-acid glycoprotein), whose concentrations increase during inflammation (Ceciliani et al., 2012; Reczyńska et al., 2018). Albumin (*ALB*) is an abundant plasma protein that is an important circulating antioxidant with ligand binding and free radical-scavenging activities (Roche et al., 2008). There is no consensus in the literature regarding albumin concentration due to heat stress in cattle since studies have described that albumin concentration increases (Berian et al., 2019; Mohapatra et al., 2021), decreases (Baek et al., 2019; Kang et al., 2019) or is unaffected by heat stress (Gaafar et al., 2021; Jo et al., 2021). In our study, albumin was found to have a lower abundance during HSP than plasma samples obtained during HSR. However, it is important to point out that all plasma samples were submitted to albumin depletion before proteomic analysis to allow the detection of the low-abundant proteins.

Clusterin (*CLU*) is a secreted chaperone that exhibits key roles in protein homeostasis, apoptosis, and prevents the aggregation and precipitation of target proteins under heat stress conditions (Carver et al., 2003; Cai et al., 2021). Clusterin is found at high concentrations in several biological fluids (Carver et al., 2003), which is regulated by many kinds of stimuli due to the combined presence of several regulatory elements that make clusterin a highly sensitive cellular biosensor of heat stress and oxidative stress (Trougakos, 2013). Calcium is an essential regulator of clusterin (Pajak and Orzechowski, 2009).

Haptoglobin (*HP*) and Hemopexin (*HPX*) are key acute phase proteins synthesized by hepatocytes and released into the circulation to bind and transport heme, playing important roles in the protection of cells from oxidative stress and the regulation of immune response (Yerbury et al., 2005; Ceciliani et al., 2012; Reczyńska et al., 2018). Haptoglobin acts as an extracellular chaperone in most body fluids, exhibiting actions similar to clusterin, inhibiting the precipitation of several proteins induced by heat or oxidation but not protecting enzymes from function loss under heat stress conditions (Yerbury et al., 2005). As reviewed by Sejian et al. (2018), haptoglobin is commonly used to assess the health and inflammatory response of animals and is considered a reliable indicator of metabolic adaptation to high heat in livestock. However, there is no consensus about the impact of hot environments on haptoglobin abundance in cattle blood plasma. Some authors did not detect the environmental temperature influence on its concentrations (Wijffels et al., 2024), while other authors detected the haptoglobin concentration decreased in animals under heat stress conditions (Kim et al., 2018), showing that heat stress does not always rises signs of acute stress indicator. In addition, several studies have been showing the plasma haptoglobin concentration increased during high temperature environments (Alberghina et al., 2013; Jo et al., 2021; Wickramasinghe et al., 2021; Koch et al., 2023), suggesting that heat stress induced an adaptive immune response in blood involving this acute-phase protein (Koch et al., 2023). As reported here, Wickramasinghe et al. (2021) and Cheng et al. (2018) also found haptoglobin levels increased in the blood plasma of dairy heifer calves and dairy cows during heat stress, respectively, which decreased significantly at the end of heat stress, returning to baseline levels, which might alleviate inflammatory response triggered by heat stress. Regarding hemopexin, a decreased abundance of its content in subcutaneous adipose tissue of late pregnant cows under high environment temperature was observed by Zachut et al. (2017), suggesting that the decreased abundances of the hemopexin and other acute-phase proteins could have caused an increase in oxidative stress. In mice, hemopexin has been described as a heat stress-responsive protein, which exhibited low abundance in the pituitary gland of mice under heat stress (Memon et al., 2016).

Serotransferrin (*TF*) participates in iron homeostasis with haptoglobin and hemopexin. Serotransferrin is mainly produced by the liver, whose main function is transporting iron ions to all proliferating cells in the body, and avoiding the toxicity of iron-mediated free radicals (Reczyńska et al., 2018). Serotransferrin was identified as upregulated in the periovulatory follicular fluid of hyperthermic cows (Rispoli et al., 2019). Alpha-1-acid glycoprotein (*ORM1*) is also a key acute-phase plasma protein produced by hepatocytes and peripheral tissues in response to inflammation that acts as a transport protein and immunomodulates innate and adaptative immunity. The alpha-1-acid glycoprotein regulation is complex because the inflammation induces an increase in its abundancy in blood serum as well as a qualitative change in its structure, which generates several glycoforms with different activities, sometimes opposite and contradictory (Ceciliani and Lecchi, 2019). Alpha-1-acid glycoprotein had their abundance reduced in jejunal mucosa from dairy cows under heat stress conditions (Koch et al., 2021), as well as in subcutaneous adipose tissue of late pregnant cows under summer heat stress (Zachut et al., 2017). However, Rispoli et al. (2013) identified the *ORM1* transcripts that were upregulated in cumulus cells surrounding the oocyte during *in vitro* maturation under heat stress.

Vitronectin (*VTN*) is a multifunctional glycoprotein that plays an important role in several biological processes and pathways such as cell adhesion, cell survivor, regulation of the coagulation pathway, activation of JNK pathway, response to heat stress, immune response, inflammation, wound healing, tissue repair, and remodeling (Schvartz et al., 1999; You et al., 2013; Sen and Ta, 2020; Goyal et al., 2023). A mammary gland proteomic study revealed an increased abundance of vitronectin in dairy cows under heat stress (Skibiel et al., 2022). Kininogen-1 (*KNG1*) acts in many physiological functions, including vasodilation and blood coagulation during inflammatory response (Damas, 1996), as well as recovery and injury triggered by heat stress (Stallings et al., 2014). Kininogen-1 was identified as upregulated in periovulatory follicular fluid (Rispoli et al., 2019), jejunal mucosa (Koch et al., 2021), and mammary gland (Skibiel et al., 2022) tissues of heat-stressed dairy cows.

The multimeric glycoprotein Von Willebrand factor (*VWF*) plays vital roles in hemostasis through platelet activation, adhesion, and aggregation and transport of several proteins in the blood, in addition to inflammatory responses and innate immunity (Kawecki et al., 2017; Drakeford et al., 2022). Proteomic analysis of adipose tissue showed a higher abundance of Von Willebrand factor in summer-calving cows when compared with winter-calving cows (Zachut et al., 2017). Mehla et al. (2014), evaluating the transcriptomic profiles in the Indian cattle (Sahiwal) blood in response to heat stress, also found the *VWF* downregulated after 24- and 48-h post-heat exposure, which suggest compromised immunity.

Apolipoprotein B-100 (*APOB*) and apolipoprotein C-III (*APOC3*) were found in high and low abundance during HSR, respectively. Apolipoprotein B-100 is part of ultra-low-density lipoproteins (ULDL) and low-density lipoproteins (LDL) that participate in cholesterol transport and lipid mobilization. At the same time, apolipoprotein C-III is a component of high-density lipoproteins (HDL), very low-density lipoproteins (VLDL), and ULDL, with roles in lipid storage and the mobilization of fat cells. The apolipoprotein B-100 levels evaluated in both blood (Basiricò et al., 2011) and liver (Basiricò et al., 2011; Shahzad et al., 2015) samples of cows during the peripartal period were deeply affected by season of calving, where cows calving in the summer had down levels in comparison to cows calving in the spring, highlighting the negative effects of hot season on apolipoprotein B-100 levels, as observed herein. Guo et al. (2022), studying the effects of late pregnant mice on the intestinal development of the fetus, showed that maternal heat stress inhibits the development of the fetal intestine, where the downregulation of the *APOC3* gene in the fetal duodenum was observed. The *APOC3* was also found to be downregulated in the *Longissimus dorsi* muscle of pigs exposed to high temperatures (Ma et al., 2019).

Alpha-2-macroglobulin (*A2M*) was detected only during HSP, while collagen alpha-1(VI) chain (*COL6A1*) was detected only during HSR. *A2M* encodes a cytokine transporter and a broad-spectrum protease inhibitor under physiological conditions, playing important roles in hemostasis, inflammation, immunity, and infections (Vandooren and Itoh, 2021; Lagrange et al., 2022), which can act as a chaperone, binding misfolded proteins and avoiding their accumulation during innate immune system activity, as well as during stress conditions (Wyatt et al., 2014), inhibiting the aggregation of heat-stressed proteins (French et al., 2008). A proteomic study with periovulatory follicle fluid of lactating dairy cows revealed that alpha -2-macroglobulin was downregulated in cows under heat stress (Rispoli et al., 2019). *COL6A1* encodes a subunit of collagen VI heterotrimer, a member of the collagen superfamily that forms the microfibrillar and fibrillar networks of the extracellular matrix and plays important roles in maintaining the integrity of several tissues. Collagen VI is found in the extracellular matrix of almost all tissues, where it is essential in inhibiting apoptosis and oxidative stress, and positively influencing autophagy, cell growth, tissue regeneration, and metabolism (Lamandé and Bateman, 2018).

## 5 Conclusion

Blood content is influenced by heat stress as a way to reduce damage by activating body hemostasis mechanisms quickly. Identifying differentially regulated proteins and their relationship and roles in critical pathways and biological processes contributes to improving the knowledge of the mechanisms behind the response to heat stress in naturally adapted cattle breeds. Additionally, proteins with high absolute log-fold change values and proteins detected only in one time point highlighted herein are potential biomarkers for early response and animal’s recovery from heat stress, such as those encoded by the *A2M, ADRA1A, COL6A1, CXADR, DGCR8, DUSP5, EPHA2, GABBR1, MAP2K7, MUC2, NEB,* and *TOPBP1* genes.

## Data Availability

The original contributions presented in the study are included in the article/Supplementary Material. The raw data cannot be made publicly available, as it is property of the Animal Science Institute and this information is commercially sensitive. Reasonable requests for access to the raw datasets for research purposes can be e-mailed to: nedeniabs@gmail.com (NBS).

## Supplementary material

SUPPLEMENTARY TABLE S1 Proteins detected in blood plasma samples of Caracu cattle only during heat stress peak (HSP).

SUPPLEMENTARY TABLE S2 Proteins detected in blood plasma samples of Caracu cattle only during heat stress recovery (HSR).

SUPPLEMENTARY TABLE S3 Proteins differentially regulated detected in blood plasma samples of Caracu cattle during heat stress peak (HSP) and heat stress recovery (HSR).

SUPPLEMENTARY TABLE S4 Gene Ontology terms and pathways revealed by functional enrichment analyses (*p* < 0.05) performed with differentially regulated proteins between heat stress peak (HSP) and heat stress recovery (HSR).

SUPPLEMENTARY TABLE S5 Protein-protein interaction network analysis of differentially regulated proteins with high confidence score containing: A. Protein annotations; B. Network coordinates; C. Nodes and degrees description; and D. Interactions.

SUPPLEMENTARY FIGURE S1 Protein-protein interaction analysis of all differentially regulated proteins between heat stress peak (HSP) and heat stress recovery (HSR). Nodes represent differentially expressed proteins (gene symbol) and lines between nodes refer to edges of confident interactions.
