# Supplementary material for: Proteomic changes of the bovine blood plasma in response to heat stress in a tropically adapted cattle breed
Source: Front Genet. 2024 Aug 1;15:1392670. doi: 10.3389/fgene.2024.1392670 (PMC11324462; doi:10.3389/fgene.2024.1392670)
Supplement: Supplementary file 1 [file Table1.docx]

**Supplementary Table S1.** Proteins detected in blood plasma samples of Caracu cattle only during heat stress peak (HSP).

| **UniProt ID** | **Protein** | **Gene symbol** | **Protein length** |
| --- | --- | --- | --- |
| A6QLA0 | Transcriptional repressor NF-X1 | *NFX1* | 1,116 |
| A7YWP4 | Histidine ammonia-lyase | *HAL* | 657 |
| G7H7V7 | Protein TOPAZ1 | *TOPAZ1* | 1,653 |
| P58126 | Potassium voltage-gated channel subfamily KQT member 3 | *KCNQ3* | 866 |
| P80416 | Cystatin-A | *CSTA* | 98 |
| Q05443 | Lumican | *LUM* | 342 |
| Q0VC89 | Palmitoyltransferase ZDHHC20 | *ZDHHC20* | 365 |
| Q1JPJ0 | NADPH-dependent diflavin oxidoreductase 1 | *NDOR1* | 597 |
| Q3MHE8 | Signal recognition particle receptor subunit alpha | *SRPRA* | 639 |
| Q7YS82 | Myoblast determination protein 1 | *MYOD1* | 318 |
| A0A3Q1LUU5 | LDL receptor related protein 12 | *LRP12* | 840 |
| A0A3Q1M429 | Uncharacterized protein | *PARD3B* | 786 |
| A0A3Q1M4X3 | Ribosomal protein S6 kinase | *RPS6KA6* | 744 |
| A0A3Q1M7D4 | Very low-density lipoprotein receptor | *VLDLR* | 805 |
| A0A3Q1MG04 | Fibrinogen beta chain ( | *FGB* | 504 |
| A0A3Q1MLF9 | Erythrocyte membrane protein band 4.1 like 1 | *EPB41L1* | 846 |
| A0A3Q1MMN6 | Integrator complex subunit 2 | *INTS2* | 1,096 |
| A0A3Q1MRG6 | Dystonin | *DST* | 3,017 |
| A0A3Q1N1W0 | CUB and Sushi multiple domains 2 | *CSMD2* | 3,310 |
| A0A3Q1NLW2 | Rho GTPase activating protein 23 | *ARHGAP23* | 1,508 |
| A0A3Q1NNQ4 | BCL2 interacting protein 1 | *BNIP1* | 139 |
| A0A452DI08 | Alpha-2-macroglobulin | *A2M* | 478 |
| A0A452DK44 | Zinc-alpha-2-glycoprotein | *AZGP1* | 299 |
| A5PJW9 | Hedgehog-interacting protein | *HHIP* | 700 |
| A7MBB6 | Vascular endothelial growth factor D | *VEGFD* | 354 |
| E1B8R5 | Poly [ADP-ribose] polymerase tankyrase-1 | *TNKS* | 1,327 |
| E1BB49 | Homeodomain interacting protein kinase 4 | *HIPK4* | 865 |
| E1BIQ3 | Syntaxin 16 | *STX16* | 322 |
| E1BIS4 | Hyperpolarization activated cyclic nucleotide gated potassium channel 4 | *HCN4* | 1,201 |
| E1BPD8 | Phosphodiesterase | *PDE11A* | 926 |
| F1MK99 | Unc-13 homolog A | *UNC13A* | 1,701 |
| F1MLP3 | ADP-ribosyl cyclase 2 | *BST1* | 318 |
| F1MMD4 | Matrix metallopeptidase 11 | *MMP11* | 491 |
| F1MN60 | Calcium-transporting ATPase | *ATP2B2* | 1,199 |
| F1MRU4 | Dynein axonemal heavy chain 3 | *DNAH3* | 3,131 |
| F1MS02 | Coiled-coil domain containing 14 | *CCDC14* | 941 |
| F1MWT0 | Alpha-mannosidase | *MAN2C1* | 1,040 |
| F1MXP6 | N-deacetylase and N-sulfotransferase 3 | *NDST3* | 522 |
| F1N261 | Tyrosine-protein kinase Lyn | *LYN* | 512 |
| F1N2H2 | SR-related CTD associated factor 11 | *SCAF11* | 1,453 |
| F1N630 | Olfactory receptor 51M1 | *OR51M1* | 320 |
| F1N693 | Homeobox protein cut-like | *CUX1* | 1,534 |
| F1N7C1 | HECT and RLD domain containing E3 ubiquitin protein ligase family member 6 | *HERC6* | 1,017 |
| G3MXG8 | Uncharacterized protein C11orf95 homolog | *ZFTA* | 490 |
| Q08D92 | Long-chain specific acyl-CoA dehydrogenase, mitochondrial | *ACADL* | 430 |
