# Supplementary material for: Proteomic changes of the bovine blood plasma in response to heat stress in a tropically adapted cattle breed
Source: Front Genet. 2024 Aug 1;15:1392670. doi: 10.3389/fgene.2024.1392670 (PMC11324462; doi:10.3389/fgene.2024.1392670)
Supplement: Supplementary file 2 [file Table2.docx]

**Supplementary Table S2.** Proteins detected in blood plasma samples of Caracu cattle only during heat stress recovery (HSR).

| **UniProt ID** | **Protein** | **Gene symbol / Ensembl ID** | **Protein length** |
| --- | --- | --- | --- |
| A0JNK3 | Serine protease HTRA2, mitochondrial | *HTRA2* | 458 |
| A1A4J7 | Protein SMG8 | *SMG8* | 999 |
| A4FUE7 | Zinc finger C2HC domain-containing protein 1A | *ZC2HC1A* | 323 |
| A5D7H3 | Choline transporter-like protein 2 | *SLC44A2* | 706 |
| A5PJC7 | IGF-like family receptor 1 | *IGFLR1* | 357 |
| A6QLL0 | Perilipin-5 | *PLIN5* | 456 |
| A7MBJ4 | Receptor-type tyrosine-protein phosphatase F | *PTPRF* | 1,898 |
| A8PU71 | Succinate dehydrogenase assembly factor 1, mitochondrial | *SDHAF1* | 118 |
| E1B7Q7 | E3 ubiquitin-protein ligase TRIP12 | *TRIP12* | 1,992 |
| F1MF74 | [F-actin]-monooxygenase MICAL2 | *MICAL2* | 1,101 |
| F1MYR9 | G protein-activated inward rectifier potassium channel 4 | *KCNJ5* | 419 |
| F1N4M2 | Myelin regulatory factor-like protein | *MYRFL* | 896 |
| G3MZC5 | AP-5 complex subunit beta-1 | *AP5B1* | 877 |
| O97831 | Adhesion G protein-coupled receptor L1 | *ADGRL1* | 1,472 |
| P00432 | Catalase | *CAT* | 527 |
| P01888 | Beta-2-microglobulin | *B2M* | 118 |
| P11024 | NAD(P) transhydrogenase, mitochondrial | *NNT* | 1,086 |
| P17599 | Synapsin-1 | *SYN1* | 706 |
| P31754 | Uridine 5'-monophosphate synthase | *UMPS* | 480 |
| P79113 | Neuropeptide Y receptor type 2 | *NPY2R* | 384 |
| P98133 | Fibrillin-1 | *FBN1* | 2,871 |
| Q06807 | Angiopoietin-1 receptor | *TEK* | 1,125 |
| Q0IIL4 | Protein kish-B | *TMEM167B* | 74 |
| Q0P569 | Nucleobindin-1 | *NUCB1* | 474 |
| Q0P5F0 | Lysosomal acid phosphatase | *ACP2* | 423 |
| Q0VCW1 | Speckle-type POZ protein | *SPOP* | 374 |
| Q1JPG1 | Radial spoke head 10 homolog B | *RSPH10B* | 840 |
| Q28019 | Latent-transforming growth factor beta-binding protein 2 | *LTBP2* | 1,842 |
| Q28142 | Neurexin-1 | *NRXN1* | 467 |
| Q32LM8 | Nurim | *NRM* | 262 |
| Q32LQ0 | Glutamyl aminopeptidase | *ENPEP* | 956 |
| Q3MI00 | DnaJ homolog subfamily B member 1 | *DNAJB1* | 340 |
| Q3SZK8 | Na(+)/H(+) exchange regulatory cofactor NHE-RF1 | *NHERF1* | 368 |
| Q58CQ9 | Pantetheinase | *VNN1* | 510 |
| Q58DV5 | 39S ribosomal protein L30 | *MRPL30* | 161 |
| Q5E947 | Peroxiredoxin-1 | *PRDX1* | 199 |
| Q5EA36 | RNA-binding protein 14 | *RBM14* | 669 |
| Q5EAE6 | Death-associated protein 1 | *DAP* | 102 |
| Q9BE39 | Myosin-7 | *MYH7* | 1,935 |
| Q9N1F0 | Inositol 1,4,5-triphosphate receptor associated 1 | *IRAG1* | 911 |
| Q9TTE1 | Serpin A3-1 | *SERPINA3-1* | 411 |
| A0A3Q1LFM6 | TBC1 domain containing kinase | *TBCK* | 893 |
| A0A3Q1LG13 | Uncharacterized protein | *VPS13B* | 3,803 |
| A0A3Q1LHW2 | Ankyrin repeat domain 42 | *ANKRD42* | 514 |
| A0A3Q1LI53 | Nectin cell adhesion molecule 1 | *NECTIN1* | 484 |
| A0A3Q1LKV0 | Immunoglobulin V-set domain-containing protein | *ENSBTAG00000052621* | 159 |
| A0A3Q1LN55 | Voltage-dependent P/Q-type calcium channel subunit alpha-1A | *CACNA1A* | 2,278 |
| A0A3Q1LQX0 | Trinucleotide repeat containing 18 | *TNRC18* | 2,910 |
| A0A3Q1LST9 | Probable RNA-binding protein 46 | *RBM46* | 485 |
| A0A3Q1LUE2 | Dmx like 1 | *DMXL1* | 3,012 |
| A0A3Q1LUS8 | Dynein heavy chain 11, axonemal | *LOC104968411* | 4,065 |
| A0A3Q1LVJ5 | Ig-like domain-containing protein | *ENSBTAG0000005016* | 130 |
| A0A3Q1LW42 | Integrator complex subunit 6 | *INTS6* | 814 |
| A0A3Q1LWJ2 | Myosin XVI | *MYO16* | 620 |
| A0A3Q1LWL2 | Protein phosphatase 1 regulatory subunit 13B | *PPP1R13B* | 1,131 |
| A0A3Q1LX44 | Crystallin beta-gamma domain containing 1 | *CRYBG1* | 2,065 |
| A0A3Q1LY31 | Uncharacterized protein | *ENSBTAG00000053071* | 2,037 |
| A0A3Q1LZN7 | Janus kinase and microtubule interacting protein 3 | *JAKMIP3* | 867 |
| A0A3Q1LZU9 | Uncharacterized protein | *ENSBTAG00000048693* | 224 |
| A0A3Q1M024 | Protocadherin 1 | *PCDH1* | 1,215 |
| A0A3Q1M0K3 | Ig-like domain-containing protein | *ENSBTAG00000051010* | 220 |
| A0A3Q1M231 | HECT-type E3 ubiquitin transferase | *NEDD4L* | 1,255 |
| A0A3Q1M3T9 | Nebulette | *NEBL* | 932 |
| A0A3Q1M766 | RAB11 binding and LisH domain, coiled-coil and HEAT repeat containing | *RELCH* | 1,220 |
| A0A3Q1M7D1 | Dynein heavy chain 12, axonemal | *ASB14* | 3,962 |
| A0A3Q1M7H1 | Shieldin complex subunit 2 | *SHLD2* | 870 |
| A0A3Q1M7H4 | Adhesion G protein-coupled receptor V1 | *ADGRV1* | 6,303 |
| A0A3Q1M7M0 | NCK associated protein 1 | *NCKAP1* | 1,068 |
| A0A3Q1M8A2 | Phosphatidylinositol-3,4,5-trisphosphate dependent Rac exchange factor 2 | *PREX2* | 1,553 |
| A0A3Q1M8L1 | Dedicator of cytokinesis 4 | *DOCK4* | 1,964 |
| A0A3Q1M9E6 | FSA_C domain-containing protein | *KIAA1109* | 4,548 |
| A0A3Q1M9I5 | Integrin beta-4 | *ITGB4* | 1,804 |
| A0A3Q1MAH3 | Endoplasmic reticulum junction formation protein lunapark | *LNPK* | 473 |
| A0A3Q1MAV9 | Collagen type VI alpha 5 chain | *COL6A5* | 2,189 |
| A0A3Q1MB17 | CAS1 domain containing 1 | *CASD1* | 967 |
| A0A3Q1MBQ9 | Ryanodine receptor 1 | *RYR1* | 4,891 |
| A0A3Q1MFA3 | Uncharacterized protein | *ENSBTAG00000049635* | 83 |
| A0A3Q1MFZ2 | Olfactory receptor | *OR13C2E* | 318 |
| A0A3Q1MI92 | Nephrocystin 1 | *NPHP1* | 672 |
| A0A3Q1MK50 | Ig-like domain-containing protein | *ENSBTAG00000054009* | 185 |
| A0A3Q1MNK0 | Diaphanous related formin 2 | *DIAPH2* | 713 |
| A0A3Q1MP73 | Serine/arginine repetitive matrix 2 | *SRRM2* | 2,545 |
| A0A3Q1MPQ5 | ALMS1 centrosome and basal body associated protein | *ALMS1* | 4,289 |
| A0A3Q1MPW6 | Protocadherin related 15 | *PCDH15* | 1,885 |
| A0A3Q1MQ75 | Oxidative stress induced growth inhibitor 1 | *OSGIN1* | 471 |
| A0A3Q1MQX4 | Protein-tyrosine-phosphatase | *PTPRQ* | 3,183 |
| A0A3Q1MRE0 | Voltage-dependent N-type calcium channel subunit alpha | *CACNA1B* | 2,158 |
| A0A3Q1MRL6 | Uncharacterized protein | *ENSBTAG00000048931* | 160 |
| A0A3Q1MRM6 | Protein mono-ADP-ribosyltransferase PARP11 | *PARP11* | 397 |
| A0A3Q1MS50 | Proline rich 14 like | *PRR14L* | 2,180 |
| A0A3Q1MUA5 | C-type lectin domain-containing protein | *LOC100294723* | 257 |
| A0A3Q1N0V0 | DUF4211 domain-containing protein | *ENSBTAG00000049577* | 306 |
| A0A3Q1N2F2 | Multidrug resistance-associated protein 4-like | *LOC100848700* | 1,227 |
| A0A3Q1N348 | Heterogeneous nuclear ribonucleoprotein H1 | *HNRNPH1* | 462 |
| A0A3Q1N3Z8 | Platelet-derived growth factor receptor beta | *PDGFRB* | 1,107 |
| A0A3Q1N4H7 | Tankyrase 1 binding protein 1 | *TNKS1BP1* | 1,774 |
| A0A3Q1N504 | TFIID subunit TAF5 NTD2 domain-containing protein | *ENSBTAG00000052771* | 571 |
| A0A3Q1N6T9 | Fibrillin 3 | *FBN3* | 2,752 |
| A0A3Q1N7L6 | Centrosomal protein 104 | *CEP104* | 941 |
| A0A3Q1NBQ6 | Protein phosphatase 6 regulatory subunit 3 | *PPP6R3* | 844 |
| A0A3Q1NCE0 | Olfactory receptor 9K2 | *OR9K2I* | 317 |
| A0A3Q1NE39 | ArfGAP with GTPase domain, ankyrin repeat and PH domain 3 | *AGAP3* | 426 |
| A0A3Q1NEW0 | Transient receptor potential cation channel subfamily V member 1 | *TRPV1* | 777 |
| A0A3Q1NI96 | Ig-like domain-containing protein | *ENSBTAG00000053626* | 622 |
| A0A3Q1NNQ8 | Integrin subunit alpha E | *ITGAE* | 1,141 |
| A0JN68 | Myelin transcription factor 1-like | *MYT1L* | 1,144 |
| A4IF67 | Zinc finger protein 200 | *ZNF200* | 393 |
| A4UAF0 | Sperm associated antigen 11 isoform C | *SPAG11* | 138 |
| A5D9E9 | Complement subcomponent C1r | *C1R* | 705 |
| A6QLN8 | SCY1-like protein 2 | *SCYL2* | 929 |
| A7Z077 | Retroelement silencing factor 1 | *RESF1* | 1,661 |
| E1B7C1 | Lymphocyte activation gene 3 protein | *LAG3* | 516 |
| E1B7F5 | WD repeat and HMG-box DNA binding protein 1 | *WDHD1* | 1,127 |
| E1B8E2 | Capicua transcriptional repressor | *CIC* | 1,603 |
| E1B8U8 | Coiled-coil and C2 domain containing 2A | *CC2D2A* | 1,565 |
| E1B949 | FAT atypical cadherin 4 | *FAT4* | 4,982 |
| E1B9N7 | ATP-dependent RNA helicase DHX29 | *DHX29* | 1,366 |
| E1B9R5 | Dynein axonemal heavy chain 8 | *DNAH8* | 4,730 |
| E1B9S9 | Voltage-dependent L-type calcium channel subunit alpha-1F | *CACNA1F* | 1,972 |
| E1BAG3 | Thioredoxin domain containing 16 | *TXNDC16* | 818 |
| E1BBD8 | Oxysterol-binding protein | *OSBPL7* | 845 |
| E1BBF0 | Serine/threonine kinase 31 | *STK31* | 1,018 |
| E1BBR9 | Protein SPT2 homolog | *SPTY2D1* | 681 |
| E1BCU7 | RNA N6-adenosine-methyltransferase METTL16 | *METTL16* | 643 |
| E1BD54 | Anion exchange transporter | *SLC26A7* | 655 |
| E1BDX1 | CST complex subunit CTC1 | *CTC1* | 1,210 |
| E1BDX8 | Dynein cytoplasmic 1 heavy chain 1 | *DYNC1H1* | 4,645 |
| E1BFF7 | MARVEL domain-containing protein | *CKLF* | 149 |
| E1BFK8 | Regulatory factor X6 | *RFX6* | 927 |
| E1BHT5 | Ubiquitin protein ligase E3 component n-recognin 4 | *UBR4* | 5,181 |
| E1BHY5 | Dickkopf WNT signaling pathway inhibitor 4 | *DKK4* | 225 |
| E1BI26 | Mediator of DNA damage checkpoint protein 1 | *MDC1* | 1,671 |
| E1BI98 | Collagen type VI alpha 1 chain | *COL6A1* | 1,027 |
| E1BIQ2 | ArfGAP with GTPase domain, ankyrin repeat and PH domain 2 | *AGAP2* | 1,188 |
| E1BK33 | Microtubule associated serine/threonine kinase 1 | *MAST1* | 1,572 |
| E1BLZ0 | Ubiquitin carboxyl-terminal hydrolase | *USP44* | 709 |
| E1BN69 | Leucine rich repeats and immunoglobulin like domains 3 | *LRIG3* | 1,119 |
| E1BN70 | Integrator complex subunit 3 | *INTS3* | 1,040 |
| E1BND1 | Kin17 DNA and RNA binding protein | *KIN* | 392 |
| E1BPE9 | KH domain containing 3 like, subcortical maternal complex member | *KHDC3L* | 240 |
| E1BPK7 | Zinc finger FYVE-type containing 1 | *ZFYVE1* | 777 |
| E1BPP1 | TTK protein kinase | *TTK* | 916 |
| F1MAZ0 | Zinc finger and BTB domain containing 1 | *ZBTB1* | 713 |
| F1MB09 | Calpain 14 | *CAPN14* | 685 |
| F1MCQ4 | Gasdermin B | *GSDMB* | 443 |
| F1MD76 | Folliculin interacting protein 2 | *FNIP2* | 1,021 |
| F1MDE6 | Amyloid beta precursor protein binding family B member 2 | *APBB2* | 757 |
| F1MDR7 | DnaJ homolog subfamily C member 13 | *DNAJC13* | 2,243 |
| F1MFC4 | Phospholipid-transporting ATPase IK | *ATP8B3* | 1,401 |
| F1MFH7 | Ephrin type-A receptor 1 | *EPHA1* | 982 |
| F1MHF0 | Membrane associated ring-CH-type finger 6 | *MARCHF6* | 910 |
| F1MHI6 | Prickle planar cell polarity protein 2 | *PRICKLE2* | 844 |
| F1MI18 | Alpha-2-macroglobulin | *LOC506828* | 1,433 |
| F1MIU1 | Uncharacterized protein | *LOC510536* | 445 |
| F1MJS9 | Receptor-type tyrosine-protein phosphatase C | *PTPRC* | 1,243 |
| F1MLB1 | E1A binding protein p400 | *EP400* | 3,092 |
| F1MLC4 | Transmembrane protein 8B | *TMEM8B* | 930 |
| F1MNA1 | Tudor domain containing 9 | *TDRD9* | 1,352 |
| F1MPW1 | Disco interacting protein 2 homolog A | *DIP2A* | 1,564 |
| F1MPW3 | Zinc finger protein 770 | *ZNF770* | 688 |
| F1MQI2 | SAP domain-containing protein | *DPPA2* | 315 |
| F1MTE4 | JRK like | *JRKL* | 524 |
| F1MTQ0 | Bromodomain and WD repeat domain containing 1 | *BRWD1* | 2,323 |
| F1MTZ4 | Fibrillin 2 | *FBN2* | 2,912 |
| F1MUS6 | Reelin | *RELN* | 3,460 |
| F1MV84 | Synaptonemal complex protein 2 | *SYCP2* | 1,336 |
| F1MWK8 | Inactive tyrosine-protein kinase 7 | *PTK7* | 1,070 |
| F1MXH6 | Mitogen-activated protein kinase kinase kinase | *MAP3K5* | 1,366 |
| F1MY45 | Carnosine synthase 1 | *CARNS1* | 951 |
| F1MY85 | Complement C5a anaphylatoxin | *C5* | 1,677 |
| F1MY99 | Olfactory receptor 18 | *OR7E188* | 326 |
| F1MYZ4 | Semaphorin 4B | *SEMA4B* | 828 |
| F1MZ31 | Tigger transposable element derived 3 | *TIGD3* | 471 |
| F1MZE4 | Sperm-associated antigen 7 | *SPAG7* | 227 |
| F1N084 | Chondroitin sulfate proteoglycan family member 4B | *CSPG4B* | 2,339 |
| F1N0B2 | Phospholipid-transporting ATPase | *LOC536660* | 1,197 |
| F1N0P9 | GID complex subunit 4 homolog | *GID4* | 217 |
| F1N163 | Ephrin-A3 | *EFNA3* | 238 |
| F1N1F1 | Codanin 1 | *CDAN1* | 1,230 |
| F1N273 | Olfactory receptor 5B12-like | *OR5B139* | 341 |
| F1N2N9 | Outer dynein arm-docking complex subunit 1 | *ODAD1* | 687 |
| F1N2Q8 | SZT2 subunit of KICSTOR complex | *SZT2* | 2,576 |
| F1N401 | Collagen alpha-1(XII) chain | *COL12A1* | 3,065 |
| F1N4K1 | Phosphoribosylformylglycinamidine synthase | *PFAS* | 1,367 |
| F1N6H1 | LDL receptor related protein 2 | *LRP2* | 4,622 |
| F6R7X9 | Coiled-coil domain containing 8 | *CCDC8* | 510 |
| F6RJL7 | TNF receptor associated factor 4 | *TRAF4* | 737 |
| F6RT22 | Hyaluronan binding protein 4 | *HABP4* | 417 |
| G3MX06 | Ig-like domain-containing protein | *ENSBTAG00000045514* | 124 |
| G3MX80 | Olfactory receptor 52D1-like | *OR52S27* | 320 |
| G3MYH9 | Interferon alpha-B-like | *LOC618947* | 189 |
| G3N3N2 | Calcium voltage-gated channel subunit alpha1 C | *CACNA1C* | 837 |
| G3X6D0 | FERM and PDZ domain containing 3 | *FRMPD3* | 1,738 |
| G5E5L9 | IQ motif and Sec7 domain ArfGEF 3 | *IQSEC3* | 1,183 |
| G5E5Q5 | Patched domain containing 1 | *PTCHD1* | 888 |
| G5E604 | Ig-like domain-containing protein | *ENSBTAG00000039237* | 123 |
| G5E677 | Serine/threonine kinase 32A | *STK32A* | 366 |
| G5E6C7 | Olfactory receptor family 10 subfamily K member 1 | *OR10K1* | 315 |
| G5E6L5 | Centrosomal protein 290 | *ENSBTAG00000051348* | 807 |
| Q0P592 | Protein-ribulosamine 3-kinase | *FN3KRP* | 309 |
| Q1RMW4 | DCC-interacting protein 13-beta | *APPL2* | 662 |
| Q3ZC51 | SS18L2 protein | *SS18L2* | 77 |
