# Supplementary material for: Proteomic changes of the bovine blood plasma in response to heat stress in a tropically adapted cattle breed
Source: Front Genet. 2024 Aug 1;15:1392670. doi: 10.3389/fgene.2024.1392670 (PMC11324462; doi:10.3389/fgene.2024.1392670)
Supplement: Supplementary file 3 [file Table3.docx]

**Supplementary Table S3.** Proteins differentially regulated detected in blood plasma samples of Caracu cattle during heat stress peak (HSP) and heat stress recovery (HSR).

| **UniProt ID** | **Protein** | **Gene symbol / Ensembl ID** | **Protein length** | **LogFC^1^** |
| --- | --- | --- | --- | --- |
| A0A3Q1MBN0 | Gamma-aminobutyric acid type B receptor subunit 1 | *GABBR1* | 955 | -9.073 |
| E1BLN1 | Synaptonemal complex protein 1 | *SYCP1* | 915 | -8.035 |
| E1BMN7 | Ankyrin repeat and BTB/POZ domain-containing protein 2 | *ABTB2* | 1,191 | -7.821 |
| F1MQM7 | Dual specificity protein phosphatase 5 | *DUSP5* | 457 | -6.798 |
| A0A3Q1M2T0 | Mucin-2 | *MUC2* | 3,203 | -6.175 |
| Q28021 | Rho-associated protein kinase 2 | *ROCK2* | 1,388 | -5.911 |
| A0A3Q1N1L9 | F-box/LRR-repeat protein 19 | *FBXL19* | 1,020 | -5.701 |
| Q8WMV3 | Coxsackievirus and adenovirus receptor homolog | *CXADR* | 365 | -5.272 |
| A0A3Q1LS24 | ATP-binding cassette sub-family B member 5 | *ABCB5* | 1,270 | -4.741 |
| A0A3Q1LQW4 | DNA topoisomerase 2-binding protein 1 | *TOPBP1* | 1,434 | -4.739 |
| F1MT60 | Nebulin | *NEB* | 6,888 | -4.342 |
| Q28046 | Scinderin | *SCIN* | 715 | -4.328 |
| Q3SZS9 | Ring finger protein 38 | *RNF38* | 432 | -3.990 |
| F1MQ87 | DENN domain containing 5B | *DENND5B* | 1,274 | -3.801 |
| G3N022 | Striated muscle enriched protein kinase | *SPEG* | 3,280 | -3.591 |
| E1BH40 | E3 ubiquitin-protein ligase | *MYCBP2* | 4,553 | -3.194 |
| E1BG04 | Otoconin 90 | *OC90* | 489 | -3.076 |
| E1BDG2 | Sorting nexin 31 | *SNX31* | 437 | -3.033 |
| G3N277 | Kinesin-like protein | *KIF2A* | 744 | -3.025 |
| E1B8S9 | Palmitoyltransferase | *ZDHHC8* | 753 | -3.007 |
| A0A3Q1LLU1 | Von Willebrand factor | *VWF* | 2,810 | -2.878 |
| Q28156 | cGMP-specific 3',5'-cyclic phosphodiesterase | *PDE5A* | 865 | -2.860 |
| Q2TBH0 | Outer dense fiber protein 3 | *ODF3* | 254 | -2.844 |
| O62830 | Protein phosphatase 1B | *PPM1B* | 484 | -2.686 |
| E1BIN5 | Cullin 3 | *CUL3* | 768 | -2.555 |
| A0A3Q1MA45 | SRCR domain-containing protein | *ENSBTAG00000050618* | 433 | -2.531 |
| A6QPA6 | Myosin-3 | *MYH3* | 1,940 | -2.425 |
| P62285 | Abnormal spindle-like microcephaly-associated protein homolog | *ASPM* | 3,371 | -2.374 |
| A6H7I3 | Protein angel homolog 2 | *ANGEL2* | 544 | -2.272 |
| A6QNT8 | Protein transport protein Sec24A | *SEC24A* | 1,099 | -2.152 |
| A0A3Q1LMS2 | Spermatogenesis associated 5 | *SPATA5* | 819 | -2.105 |
| E1BP05 | Usherin | *USH2A* | 5,204 | -2.097 |
| F1MVQ7 | Uncharacterized protein | *LOC507550* | 618 | -2.087 |
| P33433 | Histidine-rich glycoprotein | *HRG* | 396 | -2.073 |
| Q3MHN2 | Complement component C9 | *C9* | 548 | -2.065 |
| Q3ZBS7 | Vitronectin | *VTN* | 476 | -2.041 |
| G3MZB1 | Dentin sialophosphoprotein | *DSPP* | 1,190 | -2.039 |
| Q3Y5Z3 | Adiponectin | *ADIPOQ* | 240 | -1.974 |
| P21214 | Transforming growth factor beta-2 proprotein | *TGFB2* | 414 | -1.960 |
| E1BNA9 | Zinc finger ZZ-type and EF-hand domain-containing protein 1 | *ZZEF1* | 2,962 | -1.928 |
| Q28065 | C4b-binding protein alpha chain | *C4BPA* | 610 | -1.903 |
| D7GLD0 | Mitofusin 2 | *MFN2* | 757 | -1.890 |
| A0A3Q1LSG0 | Keratin 42 | *KRT42* | 453 | -1.886 |
| G3X6H0 | Centrosomal protein 170B | *CEP170B* | 1,613 | -1.854 |
| P02672 | Fibrinogen alpha chain | *FGA* | 615 | -1.849 |
| Q2KJ33 | LIM and senescent cell antigen-like-containing domain protein 2 | *LIMS2* | 341 | -1.781 |
| A5PK06 | Serine/threonine-protein kinase ICK | *CILK1* | 628 | -1.732 |
| F1MNV5 | Kininogen-1 | *KNG1* | 436 | -1.722 |
| O97725 | NADH dehydrogenase [ubiquinone] 1 alpha subcomplex subunit 12 | *NDUFA12* | 145 | -1.682 |
| P19035 | Apolipoprotein C-III | *APOC3* | 96 | -1.624 |
| Q7YQE1 | Beta-1,3-galactosyl-O-glycosyl-glycoprotein beta-1,6-N-acetylglucosaminyltransferase 3 | *GCNT3* | 440 | -1.600 |
| A0A3Q1MBR6 | Uncharacterized protein | *ENSBTAG00000053696* | 264 | -1.557 |
| Q3SZR3 | Alpha-1-acid glycoprotein | *ORM1* | 202 | -1.530 |
| P02676 | Fibrinogen beta chain | *FGB* | 468 | -1.520 |
| Q58D62 | Fetuin-B | *FETUB* | 387 | -1.464 |
| F6Q4I8 | Inka box actin regulator 1 | *INKA1* | 337 | -1.452 |
| Q3ZBX6 | Large ribosomal subunit protein uL3m | *MRPL3* | 348 | -1.416 |
| F1N4I7 | Decapping mRNA 1A | *DCP1A* | 579 | -1.394 |
| F1MHR5 | Zinc finger MYM-type containing 4 | *ZMYM4* | 1,518 | -1.391 |
| Q2TBQ1 | Coagulation factor XIII B chain | *F13B* | 661 | -1.373 |
| G3X6N3 | Serotransferrin | *TF* | 704 | -1.365 |
| F1MQI5 | Fibroblast growth factor receptor like 1 | *FGFRL1* | 501 | -1.363 |
| A0A3Q1N064 | Fibrinogen gamma-B chain | *FGG* | 426 | -1.350 |
| P12799 | Fibrinogen gamma-B chain (Gamma') | *FGG* | 444 | -1.335 |
| G5E5V1 | Ig-like domain-containing protein | *ENSBTAG00000047529* | 136 | -1.292 |
| A0A3Q1M4N6 | Myosin IIIB | *MYO3B* | 1,231 | -1.269 |
| F1MLW7 | Ig-like domain-containing protein | *ENSBTAG00000050062* | 139 | -1.264 |
| G5E5C0 | Inverted formin 2 | *INF2* | 523 | -1.262 |
| A0A3Q1LMV9 | Uncharacterized protein | *EPPK1* | 3,449 | -1.260 |
| A0A3Q1LYA4 | Collagen type XXVIII alpha 1 chain | *COL28A1* | 1,034 | -1.226 |
| A0A3Q1LPC5 | Kinesin family member 16B | *KIF16B* | 1,309 | -1.213 |
| P00743 | Coagulation factor X | *F10* | 492 | -1.208 |
| F1N0W4 | Double C2-like domain-containing protein | *DOC2B* | 412 | -1.172 |
| E1BF42 | Meiosis 1 associated protein | *M1AP* | 534 | -1.168 |
| A4FUW8 | Thioredoxin domain-containing protein 11 | *TXNDC11* | 957 | -1.166 |
| G3N1H5 | Ig-like domain-containing protein | *LOC100300716* | 167 | -1.160 |
| A0A452DHX8 | Amine oxidase (EC 1.4.3.-) | *LOC100138645* | 787 | -1.148 |
| P01017 | Angiotensinogen | *AGT* | 476 | -1.145 |
| A6QLN7 | Flavin-containing monooxygenase 5 | *FMO5* | 533 | -1.102 |
| P00978 | Protein AMBP | *AMBP* | 352 | -1.091 |
| A6QPQ2 | Serpin A3-8 | *SERPINA3-8* | 418 | -1.077 |
| P07589 | Fibronectin | *FN1* | 2,478 | -1.044 |
| Q2TBU0 | Haptoglobin | *HP* | 401 | -1.039 |
| Q1RMX3 | Bcl-2-like protein 2 | *BCL2L2* | 193 | -1.002 |
| Q3SZV7 | Hemopexin | *HPX* | 459 | 1.013 |
| A6H773 | Transmembrane protein 70, mitochondrial | *TMEM70* | 254 | 1.026 |
| O02811 | Phosphatidylinositol 4-kinase alpha | *PI4KA* | 2102 | 1.143 |
| A6QNW7 | CD5 antigen-like | *CD5L* | 451 | 1.168 |
| O46470 | Regulator of G-protein signaling 7 | *RGS7* | 469 | 1.189 |
| Q2KIX7 | Protein HP-25 homolog 1 | *ENSBTAG00000030683* | 212 | 1.197 |
| E1BP14 | Rho guanine nucleotide exchange factor 17 | *ARHGEF17* | 2152 | 1.277 |
| Q0VCM5 | Inter-alpha-trypsin inhibitor heavy chain H1 | *ITIH1* | 906 | 1.286 |
| Q1RMN9 | C4b-binding protein alpha-like | *LOC510860* | 196 | 1.311 |
| A0A3Q1LPG0 | Ig-like domain-containing protein | *ENSBTAG00000050723* | 320 | 1.381 |
| E1B8D2 | DNA_MISMATCH_REPAIR_2 domain-containing protein | *MSH5* | 832 | 1.429 |
| A0A3Q1MTF8 | LPS responsive beige-like anchor protein | *LRBA* | 2809 | 1.443 |
| E1BI79 | APC regulator of WNT signaling pathway 2 | *APC2* | 2317 | 1.460 |
| A0A140T897 | Albumin | *ALB* | 607 | 1.470 |
| A0A3Q1LQK4 | Phosphatidylinositol 3-kinase catalytic subunit type 3 | *PIK3C3* | 895 | 1.484 |
| A0A3Q1MFR4 | Apolipoprotein B | *APOB* | 4554 | 1.494 |
| A0A3Q1M8H1 | Family with sequence similarity 47 member E | *ENSBTAG00000046589* | 422 | 1.556 |
| A0A3Q1N3I9 | Ig-like domain-containing protein | *ENSBTAG00000048135* | 320 | 1.594 |
| F1MQZ0 | Reticulon-1 | *RTN1* | 930 | 1.595 |
| P34955 | Alpha-1-antiproteinase | *SERPINA1 PI* | 416 | 1.611 |
| A0A3Q1LLT0 | Ig-like domain-containing protein | *ENSBTAG00000048423* | 220 | 1.615 |
| A0A3Q1LX33 | Solute carrier family 13 member 1 | *SLC13A1* | 640 | 1.656 |
| A0A452DJA9 | Serpin A3-3 | *SERPINA3-3* | 400 | 1.704 |
| F1N7H5 | Mannose-1-phosphate guanyltransferase beta | *GMPPB* | 360 | 1.752 |
| F1MF78 | Spectrin repeat containing nuclear envelope protein 2 | *SYNE2* | 6919 | 1.765 |
| A4FV18 | GEM interacting protein | *GMIP* | 965 | 1.815 |
| A0A3Q1LSM9 | HECT-type E3 ubiquitin transferase | *HUWE1* | 4356 | 1.881 |
| F1N405 | Reticulon | *RTN4* | 1230 | 1.919 |
| F1MFT1 | R3H domain and coiled-coil containing 1 | *R3HCC1* | 459 | 1.954 |
| G3MY71 | Ig-like domain-containing protein | *ENSBTAG00000048048* | 212 | 1.954 |
| A0A3B0IZF8 | Complement C1q subcomponent subunit C | *C1QC* | 243 | 1.991 |
| A0A3Q1M3A8 | Ig-like domain-containing protein | *ENSBTAG00000054090* | 186 | 2.014 |
| P23805 | Conglutinin | *CGN1* | 371 | 2.106 |
| A5PK40 | Choline transporter-like protein 3 | *SLC44A3* | 649 | 2.123 |
| F1MLW8 | Ig-like domain-containing protein | *LOC100847119* | 233 | 2.133 |
| A0A3Q1LK49 | Inter-alpha-trypsin inhibitor heavy chain H2 | *ITIH2* | 864 | 2.138 |
| P07224 | Vitamin K-dependent protein S | *PROS1 PROS* | 675 | 2.187 |
| Q08E34 | Mitochondrial import receptor subunit TOM70 | *TOMM7xxxxxx* | 609 | 2.241 |
| G3N0V0 | Ig-like domain-containing protein | *ENSBTAG00000048135* | 326 | 2.471 |
| E1BD52 | Transmembrane protein 245 | *TMEM245* | 868 | 2.482 |
| E1BNG3 | Activating signal cointegrator 1 complex subunit 3 | *ASCC3* | 2,201 | 2.522 |
| A0A3Q1NAP3 | Bromodomain and PHD finger containing 1 | *BRPF1* | 1,213 | 2.668 |
| G3N342 | Ig-like domain-containing protein | *ENSBTAG00000047632* | 491 | 2.851 |
| F1N790 | PR/SET domain 2 | *PRDM2* | 1,665 | 2.854 |
| Q2KIU3 | Protein HP-25 homolog 2 | *ENSBTAG00000018556* | 215 | 2.906 |
| E1B9R3 | UDP-glucose glycoprotein glucosyltransferase 1 | *UGGT1* | 1,413 | 2.954 |
| Q1JQC5 | Regulator of microtubule dynamics protein 3 | *RMDN3* | 471 | 2.962 |
| A0A0A0MP92 | Serpin A3-7 | *SERPINA3-7* | 417 | 2.978 |
| Q3SYV7 | Zinc finger protein 345 | *ZNF345* | 487 | 3.008 |
| A0A3Q1ML26 | Ig-like domain-containing protein | *ENSBTAG00000054702* | 219 | 3.066 |
| G3MWT1 | Ig-like domain-containing protein | *ENSBTAG00000048423* | 211 | 3.146 |
| P17697 | Clusterin | *CLU* | 439 | 3.148 |
| A0A3Q1M5U4 | Myosin XVB | *ENSBTAG00000011713* | 1,522 | 3.306 |
| P81265 | Polymeric immunoglobulin receptor | *PIGR* | 757 | 3.322 |
| A0A3Q1M3S1 | Alsin Rho guanine nucleotide exchange factor ALS2 | *ALS2* | 1,597 | 3.355 |
| A0A3Q1LRW4 | Ig-like domain-containing protein | *ENSBTAG00000047700* | 442 | 3.664 |
| A0A3Q1NKD1 | ELKS/RAB6-interacting/CAST family member 1 | *ERC1* | 1,116 | 3.817 |
| F1N6W9 | Collagen type XVIII alpha 1 chain | *COL18A1* | 1,514 | 4.176 |
| E1B968 | Solute carrier family 7 member 2 | *SLC7A2* | 669 | 4.853 |
| Q28146 | Neurexin-1 | *NRXN1* | 1,530 | 5.428 |
| P18130 | Alpha-1A adrenergic receptor | *ADRA1A* | 466 | 5.556 |
| E1BJB4 | RPGRIP1 like | *RPGRIP1L* | 1,270 | 5.622 |
| A0A3Q1LMF7 | Mitogen-activated protein kinase kinase 7 | *MAP2K7* | 670 | 5.804 |
| A6QR44 | Microprocessor complex subunit DGCR8 | *DGCR8* | 760 | 5.873 |
| E1BJ31 | Ephrin type-A receptor 2 | *EPHA2* | 975 | 7.638 |
