# Supplementary material for: Proteomic changes of the bovine blood plasma in response to heat stress in a tropically adapted cattle breed
Source: Front Genet. 2024 Aug 1;15:1392670. doi: 10.3389/fgene.2024.1392670 (PMC11324462; doi:10.3389/fgene.2024.1392670)
Supplement: Supplementary file 4 [file Table4.docx]

**Supplementary Table S4.** Gene Ontology terms and pathways revealed by functional enrichment analyses (*p* < 0.05) performed with differentially regulated proteins between heat stress peak (HSP) and heat stress recovery (HSR).

| **Term** | **N** | ***p*** | **Proteins (UniProt Accession)** | **Gene Symbols** |
| --- | --- | --- | --- | --- |
| **Gene Ontology Biological Processes** | | | | |
| GO:0006958~complement activation, classical pathway | 12 | 1.43E-09 | G3N0V0, G3N342, G3N1H5, A0A3Q1ML26, A5D9E9, A0A3B0IZF8, A0A3Q1LKV0, Q3MHN2, Q28065, A0A3Q1LLT0, F1MY85, A0A3Q1M0K3 | *ENSBTAG00000048135, ENSBTAG00000047632, LOC100300716, ENSBTAG00000054702, C1R, C1QC, ENSBTAG00000052621, C9, C4BPA, ENSBTAG00000048423, C5, ENSBTAG00000051010* |
| GO:0050853~B cell receptor signaling pathway | 9 | 7.80E-06 | G3N0V0, F1N261, G3N342, G3N1H5, F1MJS9, A0A3Q1ML26, A0A3Q1LKV0, A0A3Q1LLT0, A0A3Q1M0K3 | *ENSBTAG00000048135, LYN, ENSBTAG00000047632, LOC100300716, PTPRC, ENSBTAG00000054702, ENSBTAG00000052621, ENSBTAG00000048423, ENSBTAG00000051010* |
| GO:0050871~positive regulation of B cell activation | 7 | 1.08E-05 | G3N0V0, G3N342, G3N1H5, A0A3Q1ML26, A0A3Q1LKV0, A0A3Q1LLT0, A0A3Q1M0K3 | *ENSBTAG00000048135, ENSBTAG00000047632, LOC100300716, ENSBTAG00000054702, ENSBTAG00000052621, ENSBTAG00000048423, ENSBTAG00000051010* |
| GO:0006910~phagocytosis, recognition | 7 | 1.37E-05 | G3N0V0, G3N342, G3N1H5, A0A3Q1ML26, A0A3Q1LKV0, A0A3Q1LLT0, A0A3Q1M0K3 | *ENSBTAG00000048135, ENSBTAG00000047632, LOC100300716, ENSBTAG00000054702, ENSBTAG00000052621, ENSBTAG00000048423, ENSBTAG00000051010* |
| GO:0006911~phagocytosis, engulfment | 7 | 1.23E-04 | G3N0V0, G3N342, G3N1H5, A0A3Q1ML26, A0A3Q1LKV0, A0A3Q1LLT0, A0A3Q1M0K3 | *ENSBTAG00000048135, ENSBTAG00000047632, LOC100300716, ENSBTAG00000054702, ENSBTAG00000052621, ENSBTAG00000048423, ENSBTAG00000051010* |
| GO:0042730~fibrinolysis | 5 | 1.41E-04 | P02672, A0A3Q1N064, P12799, P07224, A0A3Q1MG04, P02676, P33433 | *FGA, FGG, FGG, PROS1, FGB, FGB, HRG* |
| GO:0072378~blood coagulation, fibrin clot formation | 4 | 1.58E-04 | A0A3Q1N064, P12799, A0A3Q1MG04, P02676, P07589, Q2TBQ1 | *FGG, FGG, FGB, FGB, FN1, F13B* |
| GO:0010951~negative regulation of endopeptidase activity | 8 | 3.68E-04 | Q58D62, A0A452DJA9, F1MNV5, P34955, Q9TTE1, A0A0A0MP92, A6QPQ2, P33433 | *FETUB, SERPINA3-3, KNG1, SERPINA1, SERPINA3-1, SERPINA3-7, SERPINA3-8, HRG* |
| GO:0018108~peptidyl-tyrosine phosphorylation | 4 | 0.001583 | F1MFH7, E1BPP1, F1MUS6, Q06807 | *EPHA1, TTK, RELN, TEK* |
| GO:0042742~defense response to bacterium | 11 | 0.001590 | G3N0V0, G3N342, G3N1H5, A4UAF0, A0A3Q1ML26, A0A3Q1LKV0, Q2TBU0, F1MCQ4, A0A3Q1LLT0, A0A3Q1M0K3 | *ENSBTAG00000048135, ENSBTAG00000047632, LOC100300716, SPAG11B, ENSBTAG00000054702, ENSBTAG00000052621, HP, GSDMB, ENSBTAG00000048423, ENSBTAG00000051010* |
| GO:0030168~platelet activation | 5 | 0.002010 | P02672, A0A3Q1N064, P12799, A0A3Q1MG04, P02676, P33433, A0A3Q1LLU1 | *FGA, FGG, FGG, FGB, FGB, HRG, VWF* |
| GO:0020027~hemoglobin metabolic process | 3 | 0.002390 | P00978, Q3SZV7, P00432 | *AMBP, HPX, CAT* |
| GO:0007155~cell adhesion | 15 | 0.002778 | F1MWK8, Q28142, A0A3Q1MG04, A0A3Q1LLU1, Q28146, A0A3Q1LI53, A0A3Q1MPW6, F1MLC4, A0A3Q1LYA4, A0A3Q1M024, A0A452DK44, F1N401, A0A3Q1NNQ8, F1MUS6, A0A3Q1M429, P02676, P07589 | *PTK7, NRXN1, FGB, VWF, NRXN1, NECTIN1, PCDH15, TMEM8B, COL28A1, PCDH1, AZGP1, COL12A1, ITGAE, RELN, PARD3B, FGB, FN1* |
| GO:0007018~microtubule-based movement | 8 | 0.002990 | E1BDX8, A0A3Q1LPC5, F1MRU4, E1B9R5, G3N277, A0A3Q1M7D1, A0A3Q1LUS8 | *DYNC1H1, KIF16B, DNAH3, DNAH8, KIF2A, DNAH12, DNAH11* |
| GO:0007596~blood coagulation | 6 | 0.003630 | P02672, F1MNV5, A0A3Q1MAH3, P00743, P07224, A0A3Q1LLU1 | *FGA, KNG1, LNPK, F10, PROS1, VWF* |
| GO:0014068~positive regulation of PI3K signaling | 6 | 0.008926 | P01017, F1MUS6, P00432, P21214, P07589, Q06807 | *AGT, RELN, CAT, TGFB2, FN1, TEK* |
| GO:2000352~negative regulation of endothelial cell apoptotic process | 4 | 0.010811 | P02672, A0A3Q1N064, P12799, A0A3Q1MG04, P02676, Q06807 | *FGA, FGG, FGG, FGB, FGB, TEK* |
| GO:0070527~platelet aggregation | 4 | 0.012173 | P02672, A0A3Q1N064, P12799, A0A3Q1MG04, P02676, P07589 | *FGA, FGG, FGG, FGB, FGB, FN1* |
| GO:0007507~heart development | 8 | 0.012663 | P98133, F1MF74, E1B8U8, Q8WMV3, P21214, G3N3N2, P07589, Q06807 | *FBN1, MICAL2, CC2D2A, CXADR, TGFB2, CACNA1C, FN1, TEK* |
| GO:0016043~cellular component organization | 3 | 0.013411 | A7MBJ4, E1BNG3, A0A3Q1M7M0 | *PTPRF, ASCC3, NCKAP1* |
| GO:0090277~positive regulation of peptide hormone secretion | 3 | 0.013411 | P02672, A0A3Q1N064, P12799, A0A3Q1MG04, P02676 | *FGA, FGG, FGG, FGB, FGB* |
| GO:0070509~calcium ion import | 4 | 0.015180 | A0A3Q1LN55, A0A3Q1MRE0, E1B9S9, G3N3N2 | *CACNA1A, CACNA1B, CACNA1F, CACNA1C* |
| GO:0031638~zymogen activation | 4 | 0.020407 | A0A3Q1MA45, A5D9E9, Q2TBU0, A6QNW7 | *ENSBTAG00000050618, C1R, HP, CD5L* |
| GO:0051258~protein polymerization | 3 | 0.023623 | P02672, A0A3Q1N064, P12799, A0A3Q1MG04, P02676 | *FGA, FGG, FGG, FGB, FGB* |
| GO:0043010~camera-type eye development | 4 | 0.024373 | P98133, F1MTZ4, E1B8U8, G3N3N2 | *FBN1, FBN2, CC2D2A, ACNA1C* |
| GO:0007157~heterophilic cell-cell adhesion via plasma membrane cell adhesion molecules | 4 | 0.026501 | O97831, E1B949, Q8WMV3, A0A3Q1LI53 | *ADGRL1, FAT4, CXADR, NECTIN1* |
| GO:1902430~negative regulation of beta-amyloid formation | 3 | 0.027550 | F1MQZ0, F1N405 | *RTN1, RTN4* |
| GO:0007605~sensory perception of sound | 7 | 0.028535 | A0A3Q1MPW6, A0A3Q1M7H4, F1N6H1, Q3SZK8, P58126, F1MN60, E1BP05 | *PCDH15, ADGRV1, LRP2, NHERF1, KCNQ3, ATP2B2, USH2A* |
| GO:0007160~cell-matrix adhesion | 5 | 0.031518 | A0A3Q1N064, A0A3Q1M9I5, P12799, A0A3Q1MG04, P02676, P07589, Q3ZBS7 | *FGG, ITGB4, FGG, FGB, FGB, FN1, VTN* |
| GO:0034116~positive regulation of heterotypic cell-cell adhesion | 3 | 0.031718 | P02672, A0A3Q1N064, P12799, A0A3Q1MG04, P02676 | *FGA, FGG, FGG, FGB, FGB* |
| GO:0008104~protein localization | 5 | 0.033249 | F1N1F1, F1N0W4, A0A3Q1M3S1, A0A3Q1M429, A0A3Q1MTF8 | *CDAN1, DOC2B, ALS2, PARD3B, LRBA* |
| GO:1905451~positive regulation of Fc-gamma receptor signaling pathway involved in phagocytosis | 2 | 0.040112 | F1MJS9, Q1RMW4 | *PTPRC, APPL2* |
| GO:0045859~regulation of protein kinase activity | 3 | 0.040733 | Q3SZK8, A1A4J7 | *NHERF1, SMG8* |
| GO:0034765~regulation of ion transmembrane transport | 6 | 0.043873 | A0A3Q1LN55, F1MYR9, A0A3Q1MRE0, E1B9S9, P58126, G3N3N2 | *CACNA1A, KCNJ5, CACNA1B, CACNA1F, KCNQ3, CACNA1C* |
| GO:0016525~negative regulation of angiogenesis | 5 | 0.044782 | Q28021, E1BJ31, P21214, P33433, Q06807 | *ROCK2, EPHA2, TGFB2, HRG, TEK* |
| GO:0045907~positive regulation of vasoconstriction | 3 | 0.045559 | A0A3Q1N064, P18130, P12799, A0A3Q1MG04, P02676 | *FGG, ADRA1A, FGG, FGB, FGB* |
| GO:0006953~acute-phase response | 3 | 0.049583 | Q3SZR3, Q2TBU0, P07589 | *ORM1, HP, FN1* |
| GO:0045087~innate immune response | 13 | 0.049906 | P02672, Q5EA36, F1N261, G3N1H5, A0A3Q1LKV0, Q58CQ9, Q28065, A0A3Q1MG04, A0A3Q1LLT0, G3N0V0, G3N342, A0A3Q1ML26, P02676, A0A3Q1M0K3 | *FGA, RBM14, LYN, LOC100300716, ENSBTAG00000052621, VNN1, C4BPA, FGB, ENSBTAG00000048423, ENSBTAG00000048135, ENSBTAG00000047632, ENSBTAG00000054702, FGB, ENSBTAG00000051010* |
| **Gene Ontology Molecular Functions** | | | | |
| GO:0034987~immunoglobulin receptor binding | 7 | 3.40E-06 | G3N0V0, G3N342, G3N1H5, A0A3Q1ML26, A0A3Q1LKV0, A0A3Q1LLT0, A0A3Q1M0K3 | *ENSBTAG00000048135, ENSBTAG00000047632, LOC100300716, ENSBTAG00000054702, ENSBTAG00000052621, ENSBTAG00000048423, ENSBTAG00000051010* |
| GO:0004867~serine-type endopeptidase inhibitor activity | 13 | 3.97E-06 | A0A452DJA9, P34955, F1MI18, A0A0A0MP92, P33433, A0A452DI08, Q0VCM5, A0A3Q1LYA4, P00978, A0A3Q1LK49, Q9TTE1, P01017, A6QPQ2 | *SERPINA3-3, SERPINA1, LOC506828, SERPINA3-7, HRG, A2M, ITIH1, COL28A1, AMBP, ITIH2, SERPINA3-1, AGT, SERPINA3-8* |
| GO:0005524~ATP binding | 49 | 5.41E-06 | A0A3Q1LMF7, A6QPA6, A0A3Q1M4N6, F1N261, F1MRU4, A0A3Q1LWJ2, G3N277, Q9BE39, A0A3Q1M7D1, G5E677, F1MXH6, F1MN60, A0A3Q1M4X3, P17599, F1MY45, A6QLN8, F1MNA1, F1MFC4, E1BB49, F1N4K1, E1BDX8, A0A3Q1N2F2, E1B9N7, A0A3Q1LPC5, E1BK33, F1MWK8, E1B9R5, Q28021, E1BPP1, A0A3Q1MBQ9, O02811, A5PK06, A0A3Q1N3Z8, F1MLB1, A0A3Q1LMS2, E1BNG3, F1N0B2, G3N022, A0A3Q1LFM6, A0A3Q1LS24, E1BBF0, F1MFH7, A0A3Q1M5U4, A0A3Q1LQK4, A0A3Q1LUS8, E1BJ31, Q06807, E1B8D2 | *MAP2K7, MYH3, MYO3B, LYN, DNAH3, MYO16, KIF2A, MYH7, DNAH12, STK32A, MAP3K5, ATP2B2, RPS6KA6, SYN1, CARNS1, SCYL2, TDRD9, ATP8B3, HIPK4, PFAS, DYNC1H1, LOC100848700, DHX29, KIF16B, MAST1, PTK7, DNAH8, ROCK2, TTK, RYR1, PI4KA, CILK1, PDGFRB, EP400, SPATA5, ASCC3, LOC536660, SPEG, TBCK, ABCB5, STK31, EPHA1, ENSBTAG00000011713, PIK3C3, DNAH11, EPHA2, TEK, MSH5* |
| GO:0003823~antigen binding | 7 | 6.92E-06 | G3N0V0, G3N342, G3N1H5, A0A3Q1ML26, A0A3Q1LKV0, A0A3Q1LLT0, A0A3Q1M0K3 | *ENSBTAG00000048135, ENSBTAG00000047632, LOC100300716, ENSBTAG00000054702, ENSBTAG00000052621, ENSBTAG00000048423, ENSBTAG00000051010* |
| GO:0004866~endopeptidase inhibitor activity | 6 | 1.06E-05 | A0A452DI08, Q58D62, F1MNV5, F1MI18, P33433, F1MY85 | *A2M, FETUB, KNG1, LOC506828, HRG, C5* |
| GO:0008569~ATP-dependent microtubule motor activity, minus-end-directed | 6 | 2.41E-05 | E1BDX8, F1MRU4, E1B9R5, A0A3Q1M7D1, A0A3Q1LUS8 | *DYNC1H1, DNAH3, DNAH8, DNAH12, DNAH11* |
| GO:0050839~cell adhesion molecule binding | 7 | 7.86E-05 | O97831, A0A3Q1N064, A7MBJ4, F1MWK8, P12799, Q8WMV3, A0A3Q1MG04, P02676, A0A3Q1LI53 | *ADGRL1, FGG, PTPRF, PTK7, FGG, CXADR FGB, FGB, NECTIN1* |
| GO:0051959~dynein light intermediate chain binding | 6 | 3.62E-04 | E1BDX8, F1MRU4, E1B9R5, A0A3Q1M7D1, A0A3Q1LUS8 | *DYNC1H1, DNAH3, DNAH8, DNAH12, DNAH11* |
| GO:0005509~calcium ion binding | 24 | 7.06E-04 | F1MK99, E1BNA9, F1MB09, A0A3Q1MRG6, P98133, F1N0W4, A0A3Q1M7H4, Q0P569, A0A3Q1MRE0, F1MTZ4, E1B949, Q28046, A0A3Q1M7D4, A5D9E9, A0A3Q1MBQ9, A0A3Q1MQ75, A0A3Q1MPW6, Q28019, A0A3Q1M024, F1N6H1, P00743, A0A3Q1N6T9, E1BG04, P07224 | *UNC13A, ZZEF1, CAPN14, DST, FBN1, DOC2B, ADGRV1, NUCB1, CACNA1B, FBN2, FAT4, SCIN, VLDLR, C1R, RYR1, OSGIN1, PCDH15, LTBP2, PCDH1, LRP2, F10, FBN3, OC90, PROS1* |
| GO:0045505~dynein intermediate chain binding | 6 | 0.001032 | E1BDX8, F1MRU4, E1B9R5, A0A3Q1M7D1, A0A3Q1LUS8 | *DYNC1H1, DNAH3, DNAH8, DNAH12, DNAH11* |
| GO:0005102~receptor binding | 12 | 0.001055 | P02672, F1MNV5, F1N261, Q3Y5Z3, F1MJS9, P12799, A0A3Q1MG04, P33433, A0A3Q1N064, A6QLN8, Q3SZK8, P02676, P21214, P07589 | *FGA, KNG1, LYN, ADIPOQ, PTPRC, FGG, FGB, HRG, FGG, SCYL2, NHERF1, FGB, TGFB2, FN1* |
| GO:0004869~cysteine-type endopeptidase inhibitor activity | 5 | 0.001288 | Q58D62, F1MNV5, Q9TTE1, P33433, P80416 | *FETUB, KNG1, SERPINA3-1, HRG, CSTA* |
| GO:0004672~protein kinase activity | 11 | 0.001450 | A0A3Q1LMF7, A6QLN8, A0A3Q1M4N6, G3N022, A0A3Q1LFM6, F1MWK8, E1BBF0, F1MFH7, Q28021, G5E677, F1MXH6 | *MAP2K7, SCYL2, MYO3B, SPEG, TBCK, PTK7, STK31, EPHA1, ROCK2, STK32A, MAP3K5* |
| GO:0008331~high voltage-gated calcium channel activity | 4 | 0.002418 | A0A3Q1LN55, A0A3Q1MRE0, E1B9S9, G3N3N2 | *CACNA1A, CACNA1B, CACNA1F, CACNA1C* |
| GO:0051087~chaperone binding | 7 | 0.002605 | A0A140T897, Q3MI00, F1N6H1, F1MD76, A0A3Q1MG04, P02676, A0A3Q1LLU1, P07589 | *ALB, DNAJB1, LRP2, FNIP2, FGB, FGB, VWF, FN1* |
| GO:0005201~extracellular matrix structural constituent | 6 | 0.003477 | P23805, A0A3Q1LYA4, P98133, F1MTZ4, A0A3Q1N6T9, F1N6W9 | *CGN1, COL28A1, FBN1, FBN2, FBN3, COL18A1* |
| GO:0005198~structural molecule activity | 8 | 0.006038 | A0A3Q1MLF9, A0A3Q1MRG6, A0A3Q1LSG0, A0A3Q1N064, A0A3Q1LMV9, E1BG04, P12799, A0A3Q1MG04, P02676 | *EPB41L1, DST, KRT42, FGG, EPPK1, OC90, FGG, FGB, FGB* |
| GO:0003779~actin binding | 11 | 0.006435 | P17599, A0A3Q1MLF9, A0A3Q1MRG6, A0A3Q1M4N6, A0A3Q1MNK0, F1MT60, A0A3Q1LWJ2, F1MF74, Q28046, G5E5C0, A0A3Q1M5U4 | *SYN1, EPB41L1, DST, MYO3B, DIAPH2, NEB, MYO16, MICAL2, SCIN, INF2, ENSBTAG00000011713* |
| GO:0008201~heparin binding | 8 | 0.008390 | Q28019, F1MQI5, P98133, A7MBJ4, F1MJS9, P33433, P07589, A0A3Q1MFR4 | *LTBP2, FGFRL1, FBN1, PTPRF, PTPRC, HRG, FN1, APOB* |
| GO:0002020~protease binding | 6 | 0.009597 | A0A452DI08, P34955, F1MI18, P80416, A0A3Q1LLU1, P07589 | *A2M, SERPINA1, LOC506828, CSTA, VWF, FN1* |
| GO:0050661~NADP binding | 4 | 0.014676 | A6QLN7, Q1JPJ0, P00432, P11024 | *FMO5, NDOR1, CAT, NNT* |
| GO:0005516~calmodulin binding | 7 | 0.028952 | F1MK99, A0A3Q1NEW0, P62285, P58126, Q9BE39, A0A3Q1MBQ9, G3N3N2 | *UNC13A, TRPV1, ASPM, KCNQ3, MYH7, RYR1, CACNA1C* |
| GO:0004842~ubiquitin-protein transferase activity | 7 | 0.036893 | E1BIN5, A0A3Q1M231, A0A3Q1LSM9, E1B7Q7, F1N7C1, E1BHT5, A0A3Q1LY31 | *CUL3, NEDD4L, HUWE1, TRIP12, HERC6, UBR4, ENSBTAG00000053071* |
| GO:0005245~voltage-gated calcium channel activity | 4 | 0.037898 | A0A3Q1LN55, A0A3Q1MRE0, E1B9S9, G3N3N2 | *CACNA1A, CACNA1B, CACNA1F, CACNA1C* |
| GO:0004712~protein serine/threonine/tyrosine kinase activity | 10 | 0.042768 | A0A3Q1N3Z8, A0A3Q1M4X3, F1N261, E1BK33, F1MFH7, E1BPP1, Q28021, E1BJ31, F1MXH6, Q06807 | *PDGFRB, RPS6KA6, LYN, MAST1, EPHA1, TTK, ROCK2, EPHA2, MAP3K5, TEK* |
| GO:0004714~transmembrane receptor protein tyrosine kinase activity | 4 | 0.049913 | A0A3Q1N3Z8, F1MFH7, E1BJ31, Q06807 | *PDGFRB, EPHA1, EPHA2, TEK* |
| GO:0019865~immunoglobulin binding | 2 | 0.049824 | P33433, A0A3Q1LLU1 | *HRG, VWF* |
| **KEGG pathways** | | | | |
| bta04610:Complement and coagulation cascades | 17 | 2.11E-11 | P02672, F1MNV5, P34955, A5D9E9, Q3MHN2, Q28065, P12799, A0A3Q1MG04, P17697, A0A3Q1LLU1, Q2TBQ1, A0A452DI08, A0A3Q1N064, P00743, A0A3B0IZF8, P07224, P02676, F1MY85, Q3ZBS7 | *FGA, KNG1, SERPINA1, C1R, C9, C4BPA, FGG, FGB, CLU, VWF, F13B, A2M, FGG, F10, C1QC PROS1 FGB, C5, VTN* |
| bta04512:ECM-receptor interaction | 8 | 0.001653 | G3MZB1, A0A3Q1M9I5, A0A3Q1MAV9, F1MUS6, E1BI98, A0A3Q1LLU1, P07589, Q3ZBS7 | *DSPP, ITGB4, COL6A5, RELN, COL6A1, VWF, FN1, VTN* |
| bta04010:MAPK signaling pathway | 15 | 0.001994 | A0A3Q1LMF7, F1N163, A0A3Q1MRE0, E1B9S9, A7MBB6, G3N3N2, F1MXH6, F1MQM7, A0A3Q1N3Z8, A0A3Q1M4X3, A0A3Q1LN55, O62830, E1BJ31, P21214, Q06807 | *MAP2K7, EFNA3, CACNA1B, CACNA1F, VEGFD CACNA1C, MAP3K5, DUSP5, PDGFRB, RPS6KA6, CACNA1A, PPM1B, EPHA2, TGFB2, TEK* |
| bta04810:Regulation of actin cytoskeleton | 11 | 0.012652 | A0A3Q1N3Z8, F1MNV5, Q28046, A0A3Q1M9I5, E1BI79, Q3MHN2, Q28021, A0A3Q1NNQ8, A0A3Q1M7M0, F1MY85, P07589 | *PDGFRB, KNG1, SCIN, ITGB4, APC2, C9, ROCK2, ITGAE, NCKAP1, C5, FN1* |
| bta05022:Pathways of neurodegeneration - multiple diseases | 18 | 0.013369 | A0A3Q1LMF7, E1BHY5, A0A3Q1MRE0, O97725, E1B9S9, E1B9R5, A0JNK3, A0A3Q1M7D1, A0A3Q1MBQ9, G3N3N2, F1MXH6, D7GLD0, A0A3Q1M3S1, E1BPK7, E1BI79, A0A3Q1LQK4, A0A3Q1LUS8, P00432 | *MAP2K7, DKK4, CACNA1B, NDUFA12, CACNA1F, DNAH8, HTRA2, DNAH12, RYR1, CACNA1C, MAP3K5, MFN2, ALS2, ZFYVE1, APC2, PIK3C3 DNAH11, CAT* |
| bta05150:Staphylococcus aureus infection | 7 | 0.014811 | A0A3Q1LSG0, A0A3Q1N064, G3N1H5, A5D9E9, A0A3B0IZF8, P12799, F1MY85 | *KRT42, FGG, LOC100300716, C1R, C1QC, FGG, C5* |
| bta04814:Motor proteins | 10 | 0.016054 | A6QPA6, E1BDX8, A0A3Q1M4N6, A0A3Q1LPC5, A0A3Q1LWJ2, E1B9R5, G3N277, A0A3Q1M7D1, Q9BE39, A0A3Q1LUS8 | *MYH3, DYNC1H1, MYO3B, KIF16B, MYO16, DNAH8, KIF2A, DNAH12, MYH7, DNAH11* |
| bta05414:Dilated cardiomyopathy | 7 | 0.016858 | E1B9S9, G3N1H5, P01017, A0A3Q1M9I5, Q9BE39, P21214, G3N3N2 | *CACNA1F, LOC100300716, AGT, ITGB4, MYH7, TGFB2, CACNA1C* |
| bta04510:Focal adhesion | 10 | 0.017170 | A0A3Q1N3Z8, A7MBB6, A0A3Q1M9I5, A0A3Q1MAV9, Q28021, F1MUS6, E1BI98, A0A3Q1LLU1, P07589, Q3ZBS7 | *PDGFRB, VEGFD, ITGB4, COL6A5, ROCK2, RELN, COL6A1, VWF, FN1, VTN* |
| bta04022:cGMP-PKG signaling pathway | 9 | 0.017973 | F1MNV5, E1B9S9, P18130, Q28156, Q9N1F0, Q28021, Q9BE39, G3N3N2, F1MN60 | *KNG1, CACNA1F, ADRA1A, PDE5A, IRAG1, ROCK2, MYH7, CACNA1C, ATP2B2* |
| bta05017:Spinocerebellar ataxia | 8 | 0.023318 | E1B8E2, A0A3Q1LN55, Q7YS82, A0A3Q1M7D4, A0A3Q1LQK4, F1MUS6, A0A3Q1MBQ9, F1MXH6 | *CIC, CACNA1A, MYOD1, VLDLR, PIK3C3, RELN, RYR1, MAP3K5* |
| bta04151:PI3K-Akt signaling pathway | 14 | 0.035733 | F1N163, G3N1H5, A7MBB6, A0A3Q1M9I5, A0A3Q1MAV9, A0A3Q1LLU1, A0A3Q1N3Z8, G3MYH9, F1MUS6, E1BI98, E1BJ31, Q06807, P07589, Q3ZBS7 | *EFNA3, LOC100300716, VEGFD, ITGB4, COL6A5, VWF, PDGFRB, LOC618947, RELN, COL6A1, EPHA2, TEK FN1, VTN* |
| bta05165:Human papillomavirus infection | 13 | 0.037272 | A0A3Q1M9I5, A0A3Q1MAV9, A0A3Q1LLU1, A0A3Q1N3Z8, G3MYH9, A6QLA0, Q3SZK8, E1BI79, E1BHT5, F1MUS6, E1BI98, P07589, Q3ZBS7 | *ITGB4, COL6A5, VWF, PDGFRB, LOC618947, NFX1, NHERF1, APC2, UBR4, RELN, COL6A1, FN1, VTN* |
| bta05410:Hypertrophic cardiomyopathy | 6 | 0.043011 | E1B9S9, P01017, A0A3Q1M9I5, Q9BE39, P21214, G3N3N2 | *CACNA1F, AGT ITGB4, MYH7, TGFB2, CACNA1C* |
| bta05171:Coronavirus disease - COVID-19 | 11 | 0.043874 | P02672, G3N1H5, A5D9E9, Q3MHN2, P12799, A0A3Q1MG04, A0A3Q1LLU1, Q2TBQ1, G3MYH9, A0A3Q1N064, A0A3B0IZF8, P02676, F1MY85 | *FGA, LOC100300716, C1R, C9, FGG, FGB, VWF, F13B, LOC618947, FGG, C1QC FGB, C5* |
| **REACTOME pathways** | | | | |
| R-BTA-977606~Regulation of Complement cascade | 6 | 2.48E-05 | A5D9E9, A0A3B0IZF8, Q3MHN2, F1MY85, P17697, Q3ZBS7 | *C1R, C1QC, C9, C5, CLU, VTN* |
| R-BTA-166658~Complement cascade | 6 | 1.29E-04 | A5D9E9, A0A3B0IZF8, Q3MHN2, F1MY85, P17697, Q3ZBS7 | *C1R, C1QC, C9, C5, CLU, VTN* |
| R-BTA-166665~Terminal pathway of complement | 3 | 9.10E-04 | Q3MHN2, F1MY85, P17697 | *C9, C5, CLU* |
| R-BTA-114608~Platelet degranulation | 7 | 0.001344 | F1MNV5, A0A140T897, P34955, A7MBB6, P07224, P21214, P17697 | *KNG1, ALB, SERPINA1, VEGFD, PROS1, TGFB2, CLU* |
| R-BTA-76005~Response to elevated platelet cytosolic Ca2+ | 7 | 0.001448 | F1MNV5, A0A140T897, P34955, A7MBB6, P07224, P21214, P17697 | *KNG1, ALB, SERPINA1, VEGFD PROS1 TGFB2, CLU* |
| R-BTA-2129379~Molecules associated with elastic fibres | 4 | 0.004853 | P98133, F1MTZ4, P21214, Q3ZBS7 | *FBN1, FBN2, TGFB2, VTN* |
| R-BTA-8957275~Post-translational protein phosphorylation | 6 | 0.005115 | F1MNV5, A0A140T897, P34955, P98133, Q0P569, A0A3Q1LK49 | *KNG1, ALB, SERPINA1, FBN1, NUCB1, ITIH2* |
| R-BTA-418457~cGMP effects | 3 | 0.006085 | Q28156, Q9N1F0, E1BPD8 | *PDE5A IRAG1, PDE11A* |
| R-BTA-381426~Regulation of Insulin-like Growth Factor (IGF) transport and uptake by Insulin-like Growth Factor Binding Proteins (IGFBPs) | 6 | 0.006217 | F1MNV5, A0A140T897, P34955, P98133, Q0P569, A0A3Q1LK49 | *KNG1, ALB, SERPINA1, FBN1, NUCB1, ITIH2* |
| R-BTA-1566948~Elastic fibre formation | 4 | 0.007258 | P98133, F1MTZ4, P21214, Q3ZBS7 | *FBN1, FBN2, TGFB2, VTN* |
| R-BTA-109582~Hemostasis | 13 | 0.007342 | F1MNV5, A0A140T897, P34955, Q28156, A7MBB6, Q9N1F0, Q8WMV3, P17697, Q2TBQ1, P07224, P21214, Q06807, E1BPD8 | *KNG1, ALB, SERPINA1, PDE5A, VEGFD, IRAG1, CXADR, CLU, F13B, PROS1 TGFB2, TEK, PDE11A* |
| R-BTA-392154~Nitric oxide stimulates guanylate cyclase | 3 | 0.008020 | Q28156, Q9N1F0, E1BPD8 | *PDE5A, IRAG1, PDE11A* |
| R-BTA-1474244~Extracellular matrix organization | 7 | 0.014761 | F1MB09, P98133, F1MTZ4, Q05443, E1BI98, P21214, Q3ZBS7 | *CAPN14, FBN1, FBN2, LUM, COL6A1, TGFB2, VTN* |
| R-BTA-216083~Integrin cell surface interactions | 4 | 0.018142 | P98133, Q05443, E1BI98, Q3ZBS7 | *FBN1, LUM, COL6A1, VTN* |
| R-BTA-140877~Formation of Fibrin Clot (Clotting Cascade) | 3 | 0.027766 | F1MNV5, P07224, Q2TBQ1 | *KNG1, PROS1, F13B* |
| R-BTA-983170~Antigen Presentation: Folding, assembly and peptide loading of class I MHC | 3 | 0.027766 | P01888, A6QNT8 | *B2M, SEC24A* |
| R-BTA-1236977~Endosomal/Vacuolar pathway | 2 | 0.034920 | P01888 | *B2M* |
| R-BTA-1474228~Degradation of the extracellular matrix | 4 | 0.049157 | F1MB09, P98133, F1MTZ4, E1BI98 | *CAPN14, FBN1, FBN2, COL6A1* |
| R-BTA-76002~Platelet activation, signaling and aggregation | 7 | 0.049332 | F1MNV5, A0A140T897, P34955, A7MBB6, P07224, P21214, P17697 | *KNG1, ALB, SERPINA1, VEGFD, PROS1, TGFB2, CLU* |
