# Supplementary figures and images for: Proteomic changes of the bovine blood plasma in response to heat stress in a tropically adapted cattle breed
Source: Front Genet. 2024 Aug 1;15:1392670. doi: 10.3389/fgene.2024.1392670 (PMC11324462; doi:10.3389/fgene.2024.1392670)

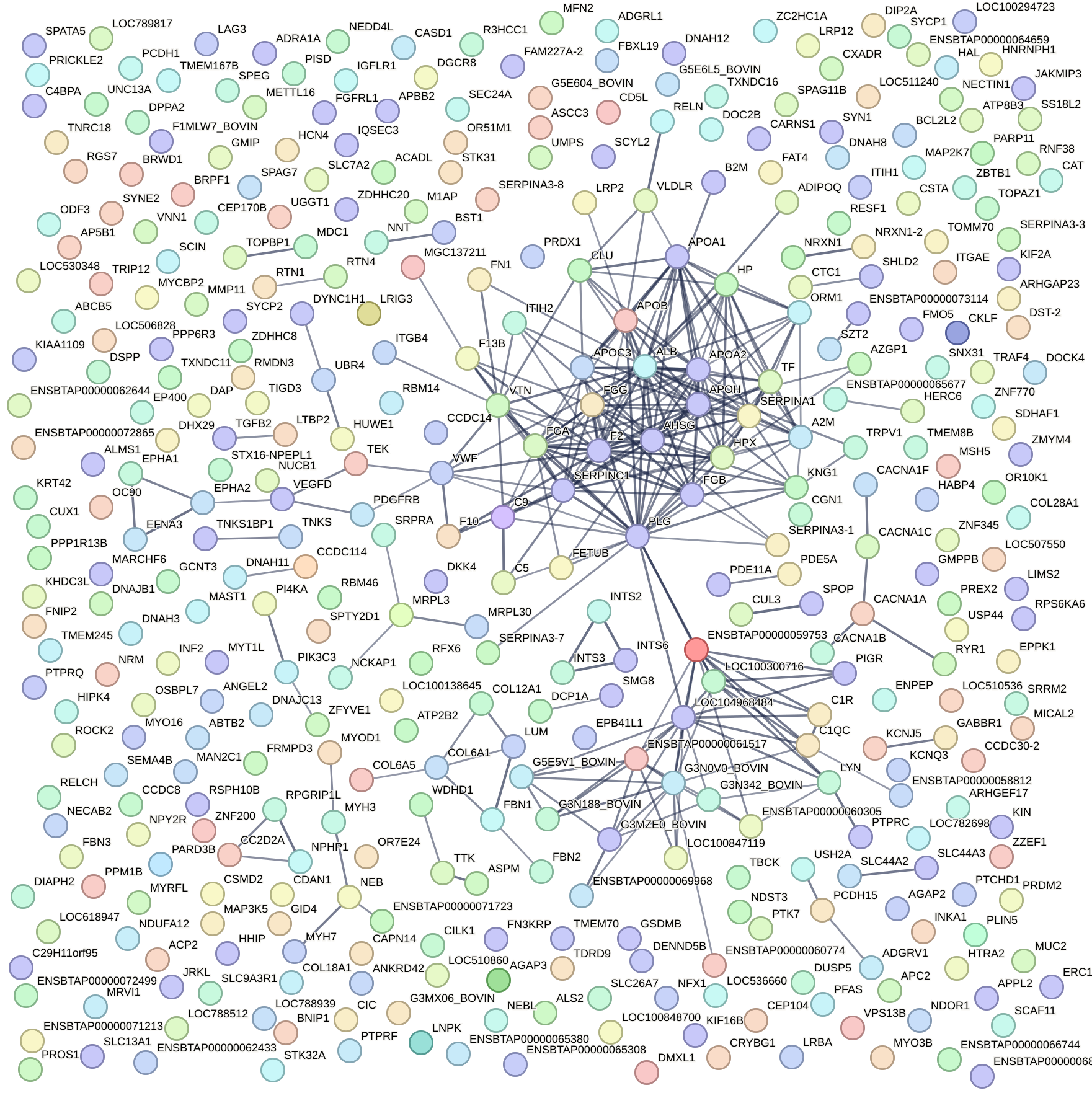

Supplement: Supplementary file 5 [file Image1.PNG]
